# Identification of novel anti-amoebic pharmacophores from kinase inhibitor chemotypes

**DOI:** 10.3389/fmicb.2023.1149145

**Published:** 2023-05-10

**Authors:** Lori Ferrins, Melissa J. Buskes, Madison M. Kapteyn, Hannah N. Engels, Suzanne E. Enos, Chenyang Lu, Dana M. Klug, Baljinder Singh, Antonio Quotadamo, Kelly Bachovchin, Westley F. Tear, Andrew E. Spaulding, Katherine C. Forbes, Seema Bag, Mitch Rivers, Catherine LeBlanc, Erin Burchfield, Jeremy R. Armand, Rosario Diaz-Gonzalez, Gloria Ceballos-Perez, Raquel García-Hernández, Guiomar Pérez-Moreno, Cristina Bosch-Navarrete, Claudia Gómez-Liñán, Luis Miguel Ruiz-Pérez, Francisco Gamarro, Dolores González-Pacanowska, Miguel Navarro, Kojo Mensa-Wilmot, Michael P. Pollastri, Dennis E. Kyle, Christopher A. Rice

**Affiliations:** ^1^Department of Chemistry and Chemical Biology, Northeastern University, Boston, MA, United States; ^2^Center for Tropical and Emerging Global Diseases, University of Georgia, Athens, GA, United States; ^3^Department of Pharmaceutical and Biomedical Sciences, College of Pharmacy, University of Georgia, Athens, GA, United States; ^4^Department of Comparative Pathobiology, College of Veterinary Medicine, Purdue University, West Lafayette, IN, United States; ^5^Clinical and Experimental Medicine PhD Program, University of Modena and Reggio Emilia, Modena, Italy; ^6^Instituto de Parasitología y Biomedicina “López-Neyra” Consejo Superior de Investigaciones Científicas (CSIC), Granada, Spain; ^7^Department of Molecular and Cellular Biology, Kennesaw State University, Kennesaw, GA, United States

**Keywords:** pathogenic free-living amoeba, *Acanthamoeba* species, *Naegleria fowleri*, *Balamuthia mandrillaris*, kinase inhibitors, cross-screening, hit-identification

## Abstract

*Acanthamoeba* species, *Naegleria fowleri*, and *Balamuthia mandrillaris* are opportunistic pathogens that cause a range of brain, skin, eye, and disseminated diseases in humans and animals. These pathogenic free-living amoebae (pFLA) are commonly misdiagnosed and have sub-optimal treatment regimens which contribute to the extremely high mortality rates (>90%) when they infect the central nervous system. To address the unmet medical need for effective therapeutics, we screened kinase inhibitor chemotypes against three pFLA using phenotypic drug assays involving CellTiter-Glo 2.0. Herein, we report the activity of the compounds against the trophozoite stage of each of the three amoebae, ranging from nanomolar to low micromolar potency. The most potent compounds that were identified from this screening effort were: **2d** (*A. castellanii* EC_50_: 0.92 ± 0.3 μM; and *N. fowleri* EC_50_: 0.43 ± 0.13 μM), **1c** and **2b** (*N. fowleri* EC_50_s: <0.63 μM, and 0.3 ± 0.21 μM), and **4b** and **7b** (*B. mandrillaris* EC_50_s: 1.0 ± 0.12 μM, and 1.4 ± 0.17 μM, respectively). With several of these pharmacophores already possessing blood–brain barrier (BBB) permeability properties, or are predicted to penetrate the BBB, these hits present novel starting points for optimization as future treatments for pFLA-caused diseases.

## Introduction

Free-living amoebas are ubiquitously found in various natural and man-made sources. Many are of no significant medical importance, but some are opportunistic pathogens of humans and animals. *Acanthamoeba* was the first potential pathogenic free-living amoebae (pFLA) described by Castellani (1930). Since then, the genus of *Acanthamoeba* has been divided into 23 genotypes (T1-T23), based on the 18S rRNA gene sequences (Diehl et al., 2021; Putaporntip et al., 2021). Of these, the most common genotype belongs to the T4 clade which has multiple species and thousands of isolates associated, these are found to cause the majority of the cases of *Acanthamoeba* keratitis (Diehl et al., 2021). Although T4 is the most prevalent genotype worldwide, T1, T3, T4, T10, and T12 have all been found to cause granulomatous *Acanthamoeba* encephalitis (GAE; Megha et al., 2018). *Acanthamoeba* can enter the body by various means, it is thought to initially infect the skin (cutaneous lesions) or respiratory tract before entering the blood circulatory system. Through hematogenous dissemination it can reach other organs and becomes a major problem when it reaches the blood–brain barrier where it can break down and enter the brain causing GAE (Marciano-Cabral and Cabral, 2003). The vast variation of genotypes, species, and clinical isolates of *Acanthamoeba* pose a significant problem with drug susceptibility variation and for treatment of the diseases that they cause. To further complicate amoeba infections and resolution of AK disease, amoebae can harbor other human pathogens where they can exchange genetic material increasing pathogenicity of both amoeba (host) and symbiont (Rayamajhee et al., 2022). Assessing the microbiome during an active infection, may provide insight into a tailored drug therapy which resolves the disease quicker without secondary infection caused by such symbiont. Further work needs to be researched to determine if specific symbionts and amoeba infections cause for concern for amoeba specific drug sensitivities and resistant profiles since we are now seeing more clinical failures and blindness with AK disease.

In 1965, *Naegleria* was first described as causing meningoencephalitis in four patients in Adelaide, Australia by Fowler and Carter (1965). Based on the 5.8S rDNA and the internal transcribed spacers (ITS; one or two) gene sequences, the genus of *Naegleria* has been identified to contain 47 different species unevenly distributed around the world (De Jonckheere, 2014). Of these 47, three have been found to cause disease in animals and one of these three, *N. fowleri* is the only species to cause the brain-eating disease in humans, primary amoebic meningoencephalitis (PAM). *Naegleria* pathology starts with contaminated water going up the nose through nasal ablution, sinus rinsing, or jumping/sliding into contaminated water sources. In the sinuses, the trophozoites are thought to use the olfactory nerve to cross the cribriform plate into the olfactory bulbs and frontal lobes of the brain where it causes major pathology (Grace et al., 2015). One of the most recent deaths in the United States from PAM came in 2021 after a child fell ill following a swim in a private pond in North Carolina (North Carolina Department of Health and Human Services, 2021).

*Balamuthia mandrillaris* is currently the only pathogenic species within the *Balamuthia* genus; it was described by Dr. G. S. Visvesvara in 1986 presenting similar encephalitis as *Acanthamoeba* GAE in a pregnant mandrill (*Mandrillus sphinx*) in San Diego Zoo Wild Animal Park, United States (Visvesvara et al., 1990). Although one study suggested *B. mandrillaris* uses a similar invasion pathway to *N. fowleri* (Kiderlen and Laube, 2004), this is not typically presented in human infection (Cope et al., 2018). Similar to *Acanthamoeba*, *B. mandrillaris* trophozoites and the persistent cyst stage, are both found in multiple infected tissues. Furthermore, in support of hematogenous dissemination, *B. mandrillaris* are typically found in clusters of patients brains close to blood vessels (Recavarren-Arce et al., 1999). Granulomatous *Acanthamoeba* Encephalitis (GAE) and *Balamuthia* Amoebic Encephalitis (BAE) are indistinguishable by diagnostic light microscopy. Recently, Next-Generation Sequencing (NGS) has proven to be a cost effective, unbiased, and quick method in the diagnosis of unknown diseases and several pFLA infections (Wang et al., 2018; Yang et al., 2019; Holmgaard et al., 2021). China has one of the fastest-growing genomics markets worldwide; it is not surprising that this is enabling earlier diagnosis of these neglected diseases. Although earlier diagnoses does not translate to curing these patients; it does open the therapeutic treatment window.

For these pFLA infections, therapeutic approaches include a multi-drug cocktail of amphotericin B and pentamidine, azoles (fluconazole, itraconazole, or posaconazole), macrolides (azithromycin), and other anti-bacterials and anti-fungals (Schuster et al., 2006; Visvesvara, 2010; Cope, 2013). Even with multi-drug cocktail treatments, CNS-involved infections are almost always fatal. The current inability to kill the trophozoites quickly, or in the case of *Acanthamoeba* and *Balamuthia* infections to inactivate the double-walled resistant cyst stage that have been found in all host infected tissues, e.g., skin, lungs, eyes, or brains, highlight the lack of effective treatments and the urgent need for developing new therapeutics against these pathogens. Given that prolonged therapy is often required, there are concerns for emergence of drug resistance (Siddiqui and Khan, 2012). Clearly, there is an unmet medical need to discover new drugs that are potent against pFLAs, are less toxic, and can cross the blood–brain barrier (BBB).

We have phenotypically screened compounds that were originally synthesized for ongoing human African trypanosomiasis and Leishmaniasis neglected tropical disease drug discovery programs (Diaz et al., 2014; Bachovchin et al., 2019) that yielded benzoxazepinoindazoles (Klug et al., 2019), pyrazolopyridazines (Tear et al., 2020), substituted azaindoles (Klug et al., 2021), and aminopurines (Singh et al., 2020). The imidazopyridines (Akao et al., 2021) were identified by the Drugs for Neglected Diseases *initiative* (DND*i*) *via* the Neglected Tropical Disease (NTD) Drug Discovery Booster project (Drugs for Neglected Diseases Initiative, 2015).

The compounds were screened against *A. castellanii, N. fowleri*, and *B. mandrillaris* logarithmic phase trophozoites. Compounds with an EC_50_ < 10 μM were classed as hits, while those between 10 and 20 μM were considered moderately potent, and compounds >20 μM were considered inactive (summarized in the Supporting Information). Of the compounds tested, seven met the hit criteria for *A. castellanii*, and an additional five were moderately potent. Against *N. fowleri*, 31 compounds met the potency criteria to be classed as a hit, and an additional 14 compounds had moderately potent activity. There were 26 hit compounds identified against *B. mandrillaris*, and another 10 compounds exhibited moderately potent activity. Compounds that yielded no activity against any of the pFLA are summarized in the Supplementary Table S1. Given the need for new hits that are potent against these pFLA, and that have demonstrated BBB exposure, this dataset provides a strong starting point for medicinal chemistry optimization.

## Materials and methods

### Maintenance of amoebae

Pathogenic *Acanthamoeba castellanii* T4 isolate (ATCC 50370) used in these studies was isolated from the eye of a keratitis patient in New York, NY, United States in 1978. This isolate was purchased from American Type Culture Collection (ATCC). Trophozoites were routinely grown axenically at 27°C in Protease Peptone-Glucose Media (PG) in non-vented 75 cm*^2^* tissue culture flasks (Olympus), until the cells were 80–90% confluent. For sub-culturing, cells were mechanically harvested to detach the cells from the culture flasks. The cells were collected by centrifugation at 3,214 RCF at 4°C. Complete PG media is prepared by the addition of 100 U/mL penicillin, and 100 μg/mL streptomycin antibiotics.

Pathogenic *Naegleria fowleri* (ATCC 30215), a clinical isolate obtained from a 9-year old boy in Adelaide, Australia, who died of PAM in 1969 was previously purchased from ATCC. Trophozoites were routinely grown axenically at 34°C, 5% CO_2_, in Nelson’s complete medium (NCM) in non-vented 75 cm*^2^* tissue culture flasks (Olympus), until the cells were 80–90% confluent. For sub-culturing, cells were placed on ice to detach the cells from the culture flasks. The cells were collected by centrifugation at 3,214 RCF at 4°C. Complete NCM media is prepared by the addition of 10% fetal bovine serum (FBS) and 100 U/mL penicillin, and 100 μg/mL streptomycin antibiotics.

Pathogenic *Balamuthia mandrillaris* (CDC:V039; ATCC 50209), a GAE isolate, isolated from a pregnant mandrill that died at the San Diego Zoo in 1986 was donated by Dr. Luis Fernando Lares-Jiménez ITSON University, Mexico. Trophozoites were routinely grown axenically in BMI media at 37°C, 5% CO_2_ in vented 75 cm*^2^* tissue culture flasks (Olympus), until the cells were 80–90% confluent. For sub-culturing, 0.25% Trypsin–EDTA (Gibco) cell detachment reagent was used to detach the cells from the culture flasks. The cells were collected by centrifugation at 3,214 RCF at 4°C. Complete BMI media is prepared by the addition of 10% FBS and 100 U/mL penicillin, and 100 μg/mL streptomycin antibiotics.

### Compound preparation

All compounds were synthesized at Northeastern University (NEU) and had purity > 95% as determined by LCMS analysis, compound identity was confirmed using ^1^H Nuclear Magnetic Resonance (NMR). The specific instruments used to fully characterize each compound are reported in the relevant reference listed in the tables, or in the Supporting Information. Compounds were supplied as powder stocks, which were reconstituted to a 20 mM stock concentration in DMSO and diluted in each amoeba’s representative neat media for standardized susceptibility screening from 20 μM.

### *In vitro* CellTiter-Glo trophocidal assay

The trophocidal activity of compounds was assessed using the CellTiter-Glo 2.0 luminescent viability assay (Promega, Madison, WI), as previously described (Rice et al., 2015, 2020a,b,c). Trophozoites were routinely cultured as described above and only logarithmic trophozoites were used. In brief, *A. castellanii*, *N. fowleri*, or *B. mandrillaris* trophozoites cultured in their corresponding complete media were seeded at 1,440, 3,000, or 16,000 cells/well into white 96-well plates (Costar 3370), respectively.

All compounds were assessed in 2-fold serial dilutions from 20 to 0.625 μM. Control wells were supplemented with 0.2% DMSO, as the negative control, or 12.5 μM of chlorhexidine as the positive control. All assays were incubated at each of the parasites’ representative growth temperatures, described above, for 72 h. At the 72-h time point, 25 μL of CellTiter-Glo 2.0 reagent was added to all wells of the 96-well plates by hand. The plates were protected from light and contents were mixed using an orbital shaker at 300 rpm at room temperature for 2 min to induce cell lysis. After shaking, the plates were equilibrated at room temperature for 10 min to stabilize the luminescent signal. The ATP luminescent signal (relative light units; RLUs) were measured at 490 nm with a SpectraMax I3X plate reader (Molecular Devices, Sunnyvale, CA, United States). The concentration of a drug that gives half-maximal response (EC_50_) were generated using total ATP RLUs where controls were calculated as the average of replicates using the Levenberg–Marquardt algorithm, using DMSO as the normalization control, as defined in CDD Vault (Burlingame, CA, United States). Values reported are from a minimum of three biological replicates with standard deviation.

### Cytotoxicity screening of reconfirmed compounds

#### Human fetal lung fibroblast (MRC5) cell assay

Intermediate plates were made as described, adding 95 μL of DMEM complete media to 5 μL of compound per well setting a 5% DMSO amount. Log-phase MRC5 cells were removed from a T-75 TC flask using TrypLE® Express (Thermo®) and dispersed by gentle pipetting. Cell density was adjusted to working concentration in prewarmed DMEM medium: 25,000 cells in 90 μL of culture were plated in 96-well transparent Nunclon plates and allowed to settle for 24 h at 37°C and 5% CO_2_. After settling incubation, 10 μL of freshly made intermediate plate were added per well: final maximal concentration for compounds was 50 μM in 0.5% DMSO per well. Plates were incubated for 48 h at 37°C and 5% CO_2_. At 4 h prior to fluorescence measurement, 20 μL of 500 μM resazurin solution was added. Fluorescence was read in an Infinite F200 plate reader (Tecan®) at 550 nm (excitation filter) and 590 nm (emission filter), as previously described (Ioset et al., 2009).

A four-parameter equation was used to fit the dose–response curves and determination of EC_50_ by SigmaPlot ® 13.0 software. Assays were performed in duplicate at least twice for positive compounds, to achieve a minimal *n* = 2 per dose response.

#### Rat skeletal muscle cell line (L6) cell assay

One hundred microliters (100 μL) per well of culture medium containing the compounds and controls were added to L6 cells previously cultured (4 × 10^3^ L6 cells per well). After 72 h at 37°C the medium was exchanged, and the viable cell number was determined by resazurin (Sigma–Aldrich) reduction. 20 μl of resazurin (1.1 mg/ml) was added to each well and incubated in the dark for 2 h at 37°C. Cell viability was estimated by using the same method as described above in the MRC5 cytotoxicity section, as previously described (Ioset et al., 2009).

## Results and discussion

### Acanthamoeba castellanii

The preliminary data for the benzoxazepinoindazole series, although limited, shows promising biological potency. Strong structure–activity relationships (SAR) dependent on *N*-alkylation was observed where alkylation of the aminopyrimidine **1c** was tolerated but not on the benzoxazepinoindazole core (**1e**; EC_50_: >20 μM). As part of our NTD optimization program, **1e** was tested at 10 mg/kg, Intraperitoneal injection (I.P.), in a pharmacokinetic (PK) model and was found to have a blood/brain ratio of 2.4 at 0.5 h and 2.1 at 4 h (Klug et al., 2019). Truncation to **1d**, with a methyl group on the indazole nitrogen, led to retention of potency against *A. castellanii*. Further analogs are needed to explore the SAR of this related chemotype which has demonstrated improved absorption, distribution, metabolism and elimination (ADME) properties, and *in vivo* exposure, though it showed decreased BBB penetration. The limited initial screen has provided promising leads meeting the hit criteria. Aqueous solubility will need to be addressed in a hit-to-lead optimization campaign.

The two most potent compounds identified against *A. castellanii* came from the pyrazolopyridazines, with **2b** being a low micromolar inhibitor and **2d** demonstrating sub-micromolar potency, though there is potent inhibition against skeletal muscle myoblast, L6 (ATCC CRL-1458), cells for both (< 0.62 μM). The aminopyrimidine of the pyrazolopyridazines is a known hinge binding motif in human kinases and the limited modifications that were tested [replacement with an aniline (**2 g**), 4-methyl aminopyrimidine (**2e**), or 2-pyridyl (**2f**) led to a loss of potency]. Compound **2b** was tested at 10 mg/kg, I.P., in a PK study and had a blood/brain ratio of 1.1 at 0.5 and 4 h (Tear et al., 2020). However, *in vivo* toxicity was observed and will be a focus of a hit-to-lead optimization campaign.

Two of the imidazopyridines tested had potency <10 μM (**3a** and **3b**) against *A. castellanii* and there is valuable SAR that can be derived from the screening set. Incorporation of the pyrazole in place of the pyridine was well tolerated and led to a moderately potent inhibitor (*cf.* **3b** and **3c**). Further, the free amine (**3a**) demonstrated improved activity versus the aliphatic (**3d**) and aromatic (**3e**)—NH derivatives. While no data is available on the BBB exposure of these compounds, the calculated Blood–Brain Barrier (BBB) Score (Gupta et al., 2019) for this series indicates potential exposure.

Azadinoles are another common motif in kinase inhibitor drug discovery. While two of the compounds tested demonstrated moderate potency versus *A. castellanii* (**4a** and **4b**) and the series had predicted brain penetration, when a related compound (**4c**) was progressed to PK there was negligible brain exposure; resolving this issue would need to be a focus of a subsequent hit-to-lead optimization campaign (Klug et al., 2021). There is some preliminary SAR that is apparent with the series, replacement of the tetrahydropyran with the piperidine (**4c**) was not tolerated and could indicate that the presence of a hydrogen bond donor at this position is unfavorable. Additionally, it is apparent that substituting the azaindole with a 4-methyl (**4b**) was well tolerated, as was introduction of the additional heteroatom (**4a**).

### Naegleria fowleri

The benzoxazepinoindazoles yielded the most potent hit for *N. fowleri* (**1c** EC_50_: <0.63 μM) which demonstrated >10-fold selectivity versus THP-1 (see Supplementary Table S1 for full data set) and MRC5 cells. Alkylation of the indazole nitrogen was unfavorable (**1e** and **1d**) though the loss of potency for **1d** may be impacted by the removal of the northern aromatic ring and further analogs are needed. The aminopyrazine motif that is present in **1a–c** leads to potent activity against *N. fowleri* and the alkylation of the amine in **1c** is well tolerated.

Modifications to the aminopyrazine of the pyrazolopyridazines led to the identification of **2f** which had potent *N. fowleri* activity (EC_50_: 1.0 μM) and no activity against MRC5 or L6 cells at 50 μM. This compound is significant as it demonstrates that alteration to the reported hinge binding region (Tavares et al., 2004; Stevens et al., 2008) and removal of one of the hydrogen bond acceptors, is tolerated, and may afford an opportunity to obtain selectivity versus human kinases. Also tolerated was replacement of the benzonitrile with the cyclohexylamine (**2p**), or 3-aminopyridine (**2b**), and substitution of the 2-position of the pyrazolopyridazine with an isopropyl (**2n**) or alkyl ether (**2 l**). While these compounds were all low micromolar versus *N. fowleri*, variable levels of toxicity were observed particularly for **2p**, **2b**, and **2d**, notably, when **2b** and **2d** (L6 CC_50_: <0.62 μM) was advanced into PK studies (dosed I.P. at 10 mg/kg) acute toxicity was seen. Given the selectivity of **2f** this serves as an excellent starting point for further optimization.

Of the imidazopyridines there were three moderately potent, or better, inhibitors of *N. fowleri*. Compound **3e** was the most potent compound with a BBB Score (5.1) that predicts BBB penetration. However, replacement of the aniline portion with aliphatic amines led to a complete loss of potency (**3f** and **3d**). Truncation of the aniline to the free amine was reasonably well tolerated (**3a**; EC_50_: 15 μM). Derivatization to the amide was unfavorable (**3k**, **3l**, and **3h**). Modification to the pyridine region was also trialed and pyrimidines (**3i** and **3j**), methylpyridine (**3c**) and 3-pyridine (**3 g**) all led to a loss of potency. Though replacement with the *N-*methylpyrazole was reasonably well tolerated (**3b**).

Of the five 2,4-disubstituted azaindoles tested, four were found to be potent against *N. fowleri* with only the amide **5e** being inactive. No modifications to the pyrazole were made, though secondary (**5d**) and tertiary (**5a** and **5c**) amines were both tolerated, as was removal of the benzylic methylene (**5b**). Of the seven 3,5-disubstituted azaindoles tested, only one was active (**4d**) and any replacement of the pyrazole led to a complete loss of potency (**4f** and **4e**). Additionally, the benzonitrile at the 3-position appears to be unfavorable for potency when comparing **4d** with **4e**.

The preliminary data for the *aminopurines*, although limited, shows moderate biological potency against *N. fowleri*, though valuable SAR data has been gleaned. Alkylation of the purine -NH (**6c**) led to a loss of activity, as did elongation (replacement with the ethylpyrrolidine; **6d**), increased bulk (4,4-difluoropiperidine; **6e**) or incorporation of heteroatoms (replaced with morpholine) into the aliphatic amine, and replacement of the thiophene with either 4-fluoroaniline or *N-*methylpyrazole amine. While we have PK data on compounds in this series, brain exposure levels were not measured and need to be assessed as part of any optimization campaign.

Only one of the lapatinib derivatives (**7a**) tested had moderate potency against *N. fowleri*. Replacement of the aryl ether with the aminopyrazine led to a complete loss of activity and, given the lipophilicity of the aryl ether the lipophilic ligand efficiency (LLE) is poor (1.40). Additional analogs with improved aqueous solubility need to be tested to confirm the activity of this series and improve our analysis.

There were 19 carbazoles tested with most of the modifications in the pendant amine. In general, the secondary amines were preferred with **8c** being more potent than **8 f**. Though, the piperidine derivative (**8d**) opposed this trend and was equipotent with **8c**. There may also be a shape and steric component to the secondary amines with **8a** showing optimal activity (EC_50_: 5.1 μM; LLE: 1.9) versus the cyclopentane (**8 k**; LLE: 1.95) and cycloheptane (**8 h**; LLE: 1.1). Two compounds from this series have previously been advanced into PK studies and **8c** was found to have excellent exposure in the brain and, at 40 mg/kg per os (P.O.; oral administration), it had a brain:blood ratio at 1 h of 5.1 (male) and 4.1 (female); and at 4 h it was 3.6 (male) and 5.2 (female; Singh et al., 2023). Further SAR scoping with a focus on reducing the lipophilicity and maintaining the potency is underway.

### Balamuthia mandrillaris

Alkylation of the indazole -NH for the **benzoxazepinoindazoles** led to a compound with moderate potency against *B. mandrillaris* (**1e**; EC_50_: 14 ± 0.29 μM). Additionally, the pyrimidine regioisomers appear to have an impact on the activity of the compounds (*cf.* **1a** and **1b**). Preliminary SAR suggests that the 2-pyridyl-5-amino motif may be driving the potency against *B. mandrillaris*. Finally, the truncated analog **1d** demonstrated improved potency (EC_50_: 9.49 ± 0.23 μM) particularly when compared to **1e** (which retains the same aminopyrazine motif).

Differential SAR was observed for the pyrazolopyridazines with the *m*-benzonitrile not well tolerated against *B. mandrillaris* (*cf.* **2c**), though this trend is complicated due to a lack of match pairs. Removal of either nitrogen in the pyrimidine led to a loss of activity (**2 g** and **2f**) which is largely consistent with the SAR observed across the other pFLA. Replacement of the aniline with the 4-aminocyclohexanol was generally not well tolerated (**2 k**), though activity could be recovered *via* substitution of the 6-methoxy (*cf.* **2 s** and **2u**) which led to potent activity against *B. mandrillaris*.

For the 2,4-disubstituted azaindoles, one compound demonstrated potent activity against *B. mandrillaris* (**5e**), however, this also had high L6 toxicity which would prevent their progression forward. Moving to the 3,5-disubstituted azaindoles led to the identification of **4b** which demonstrated potent activity and was not toxic against MRC5 cells. However, **4b** has a moderate predicted BBB penetration from the BBB Score (3.2). As opposed to the SAR that was observed for *N. fowleri* the benzonitrile substituent is preferred (**4e, 4a**, and **4b**). Replacement of the methyl on the pyrazole is tolerated with the tetrahydropyran (**4a** and **4b**), but not the piperidine (**4c**, Table 1) suggesting that a basic nitrogen may not be tolerated.

**Table 1:** Active compounds against *Acanthamoeba castellanii*, *n* = 3.

Class	ID	Structure	*Acanthamoeba castellanii*	MRC5	L6 CC_50_
			EC_50_ (μM) ± SD	CC_50_ (μM) ± SD	(μM) ± SD
Benzoxazepinoindazoles (Klug et al., 2019)	**1a**	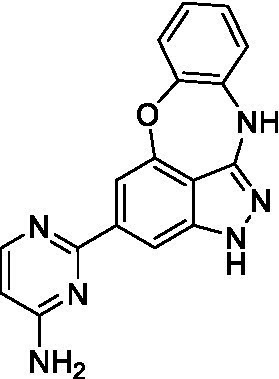	3.8 ± 0.46	> 50	32 ± 2.5
	**1b**	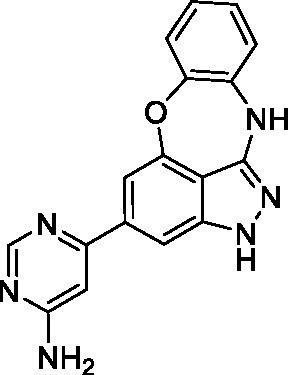	12 ± 8.2	> 50	1.0 ± 0.14
	**1c**	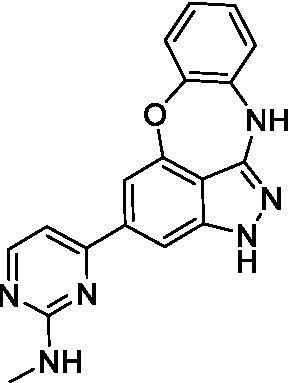	3.3 ± 0.31	> 50	6.3 ± 0.55
	**1d**	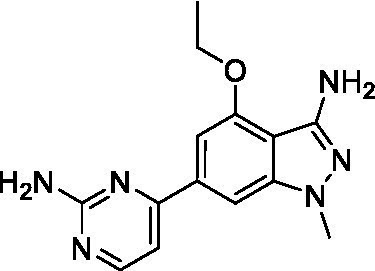	12 ± 8.3	> 50	> 17 ± 0.05
	**1e**	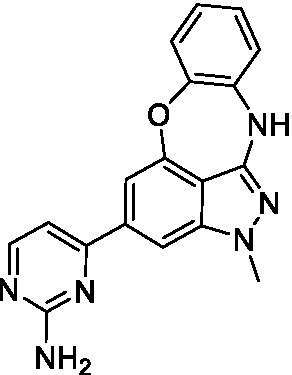	> 20	> 50	> 50
Pyrazolopyridazines (Tear et al., 2020)	**2a**	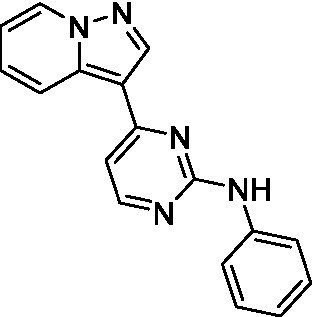	13 ± 6.9	> 50	3.2 ± 0.08
	**2b**	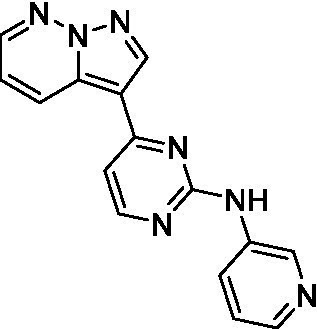	1.5 ± 0.12	> 50	< 0.62
	**2c**	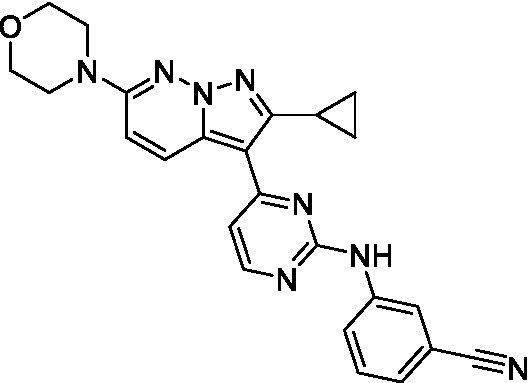	5.9 ± 0.17	> 50	22 ± 2.1
	**2d**	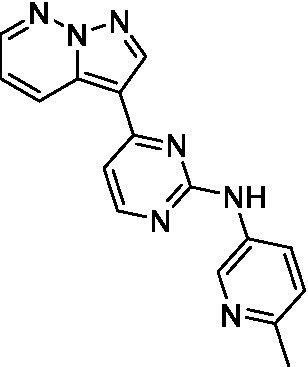	0.92 ± 0.3	> 50	< 0.62
	**2e**	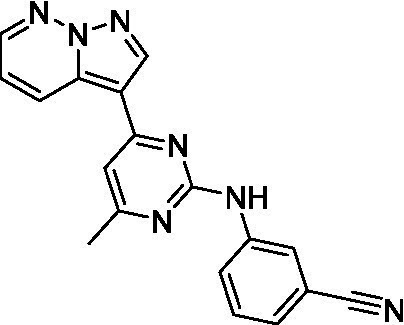	> 20	> 50	> 50
	**2f**	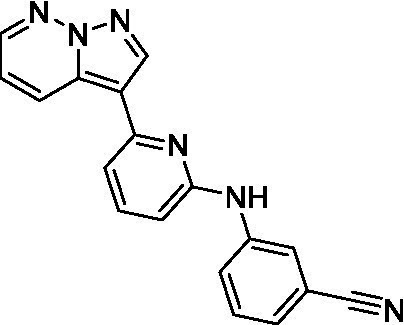	> 20	> 50	> 50
	**2 g**	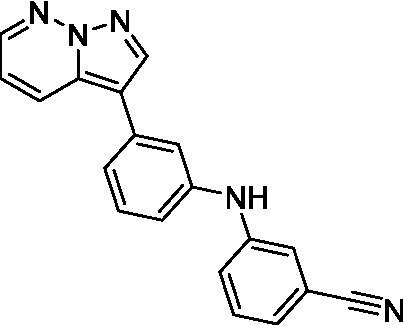	> 20	19 ± 1.0	28 ± 1.1
Imidazopyridines (Dichiara et al., 2022)	**3a**	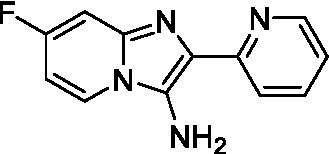	6.94 ± 0.11	*nt*	*nt*
	**3b**	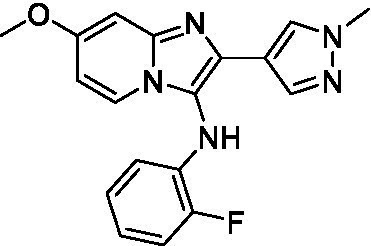	4.33 ± 0.57	*nt*	*nt*
	**3c**	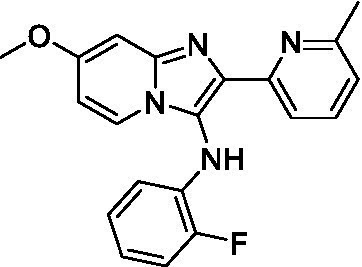	> 20	*nt*	*nt*
	**3d**	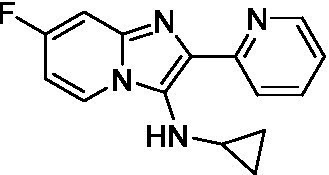	> 20	*nt*	*nt*
	**3e**	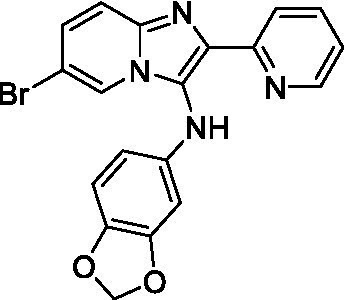	> 20	*nt*	*nt*
3-5-substituted azaindoles (Klug et al., 2021)	**4a**	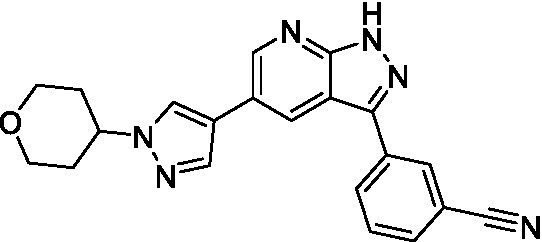	11 ± 9.0	> 50	2.8 ± 0.92
	**4b**	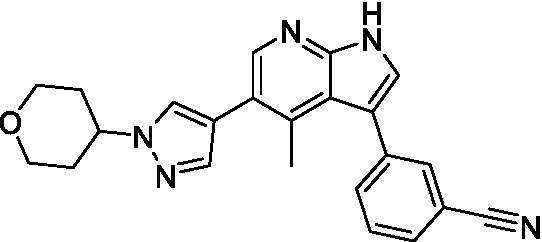	11 ± 2.3	> 50	11 ± 1.5
	**4c**	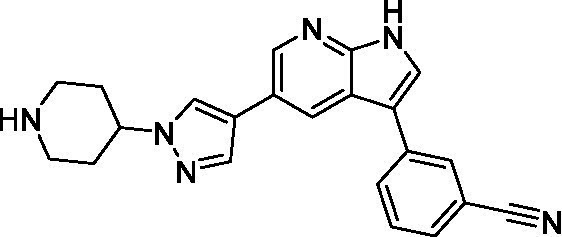	> 20	13 ± 2.4	9.0 ± 0.78
Reference Drugs	Azithromycin		0.26 ± 0.14		
	Chlorhexidine		11 ± 3.5		
	Pentamidine		4.2 ± 0.08		

nt, not tested.

Only one of the lapatinib derivatives (**7b**) tested was active against *B. mandrillaris*, and it showed potent inhibition. Compound **7b** is structurally related to several analogs in the data set and its activity could be driven by either a change in the lipophilicity (though this would be negligible when compared to the other bridged piperazines, or spirocycles), or due to the change in vector. Further analogs are required to try and understand the specifics of the observed SAR.

The SAR for the carbazoles largely tracks with that observed for *N. fowleri* with **8c** being the most active compound against *B. mandrillaris* (EC_50_: 2.4 μM). Though there are some notable differences; for example, the homomorpholine (**8j**) was well tolerated (EC_50_: 3.3 μM), and the (R)-methylmorpholine derivative (**8 l**) showed moderate inhibition (EC_50_: 12 μM) which could be due to the configuration of the methyl group.

### Current anti-amoebic remedies, and the drug discovery landscape

No standardized treatment regimens or FDA compounds are approved for *Acanthamoeba* CNS infections with the recommended treatments being empirically based on previous reports of the very limited number of patients that have survived GAE. As is true with other free-living amoeba infections, therapeutic approaches include the use of a multi-drug cocktail of antimicrobials (amphotericin B and pentamidine), azoles (fluconazole or itraconazole), macrolides (azithromycin), and, recently, miltefosine (Schuster et al., 2006; Visvesvara, 2010; Cope, 2013). Even with the laborious use of multi-drug cocktail-based treatments, CNS-involved infections are almost always fatal possibly due to the likelihood of inducing encystation. The current inability to kill the double-walled resistant cyst stage that have been found in all host infected tissues, e.g., skin, lungs, eye(s), or brain, and emphasizes the lack of effective treatments and the urgent need for developing new therapeutics against this pathogen. Besides from 5-fluorocytosine being rigorously tested in *in vivo* models of *Acanthamoeba* GAE in 1974 (Stevens and O’dell, 1974) there has not been any dedicated efforts in this space. We have previously identified repurposed drugs with a demonstrated potential for therapeutic use but many of these are not known to cross the BBB and will require significant optimization (Rice et al., 2020a). Therefore, compounds **1a**, and **1c** from the benzoxazepinoindazole series, **2b**, **2c**, and **2d** from the pyrazolopyridazine series, and **3a** and **3b** from the imidazopyridine series would suggest utility and further development of these pharmacophores against *Acanthamoeba*.

Of the CDC recommended multi-drug combination therapeutics suggested for *Naegleria fowleri* CNS disease, PAM, only amphotericin B, posaconazole, and azithromycin displayed great nanomolar activity (Colon et al., 2018). Fluconazole and miltefosine both display micromolar activity (Troth and Kyle, 2021). We note that several CYP51 inhibitors have previously been reported with low nanomolar-to-micromolar activity against *N. fowleri*, and acceptable cLogP values, though there is no measure of BBB penetration potential (Debnath et al., 2017). Separately, compounds which were predicted to have BBB penetration properties through *in vivo* models such as, posaconazole, ketoconazole, corifungin, rokitamycin, and roxithromycin have demonstrated varying level of curative effects for PAM resolution (Debnath et al., 2012; Debnath, 2021). Though there has not been any reported follow up on these compounds and the addition of compounds that will likely have a different mechanism of action would be advantageous. Therefore, compounds with EC_50_ ≤ 14 μM (better than fluconazole) will be prioritized for hit-to-lead optimization. This would include the benzoxazepinoindazoles (**1a**, **1b**, and **1c**), pyrazolopyridazines (**2a-d**, **2f**, **2 h**, **2j**, **2 k**, 2**l**, **2n-q**, and **2 s**), 3-5-substituted azaindoles (**4d**), 2,4-substituted azaindoles (**5a–d**), and carbazoles (**8a–d**, **8 g**, **8h**, and **8j-k**) from this study.

Currently, no effective treatments for infections caused by *B. mandrillaris* have been identified, instead a cocktail of drugs has been recommended by the CDC based on previous reports of successful therapeutic intervention (Centers for Disease Control and Prevention, 2021). We have previously tested all compounds within the CDC’s suggested treatment regimen (Phan et al., 2020). Of these active therapeutics, only pentamidine, flucytosine, and chlorpromazine are known to cross the BBB yet CNS-involved infections are almost always fatal, emphasizing the lack of effective treatments and the urgent need for developing new therapeutic approaches. We define any compound with ≥2x better activity than pentamidine (EC_50_ = 18.35 μM) would warrant further exploration, therefore, the benzoxazepinoindazoles (**1b**, **1c**, and **1d**), pyrazolopyridazines (**2a**, **2b**, **2c**, **2 h**, **2j**, **2p**, and **2u**), 3-5-substituted azaindoles (**4a-b**, and **4e**), 2,4-substituted azaindoles (**5b** and **5e**), 4-aminoquinolines (**7b**), and carbazoles (**8a**, **8c**, **8 h-k**) will be prioritized against *B. mandrillaris* in our future hit-to-lead optimization.

With a dearth of ongoing drug discovery efforts for anti-amoebics we believe that the described results (Tables 1–3) represent a significant contribution to this community. These compounds demonstrate superior activity in comparison to currently used anti-amoebic therapies. This, coupled with predicted, or measured BBB exposure in mice, establish this data set as highly unique. Hit-to-lead optimization efforts are underway for several of these chemotypes against the respected pathogenic free-living amoebae and we will report the results of this in future communications.

**Table 2:** Active compounds against *Naegleria fowleri*, *n* = 3.

Class	ID	Structure	*Naegleria fowleri*	MRC5 CC_50_	L6 CC_50_
			EC_50_ (μM) ± SD	(μM) ± SD	(μM) ± SD
Benzoxazepinoindazoles (Klug et al., 2019)	**1a**	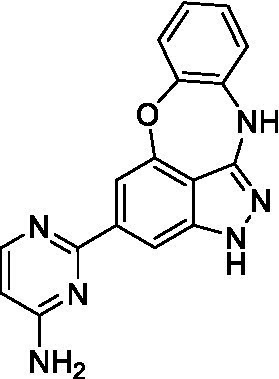	8.5 ± 0.34	> 50	32 ± 2.5
	**1b**	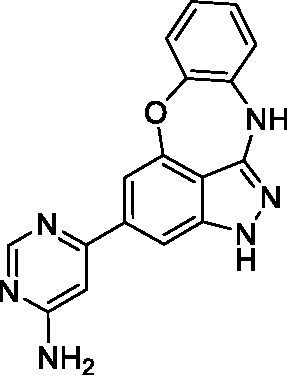	3.9 ± 0.62	> 50	1.0 ± 0.14
	**1c**	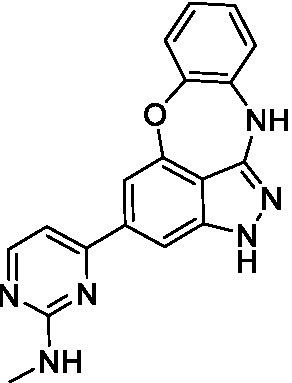	< 0.63	> 50	6.3 ± 0.55
	**1d**	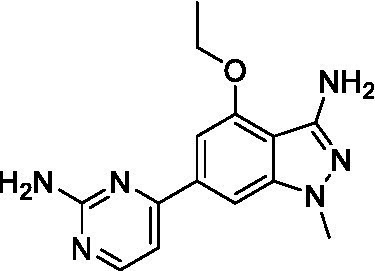	> 20	> 50	> 17 ± 0.05
	**1e**	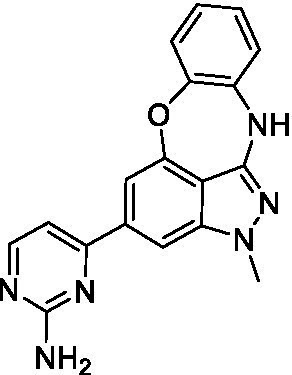	> 20	> 50	> 50
Pyrazolopyridazines (Tear et al., 2020)	**2a**	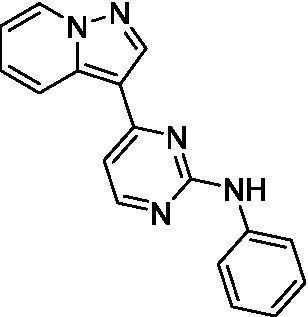	0.94 ± 0.17	> 50	3.2 ± 0.08
	**2b**	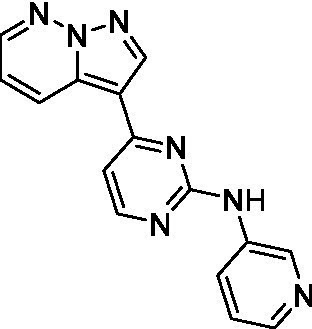	0.3 ± 0.21	> 50	< 0.62
	**2c**	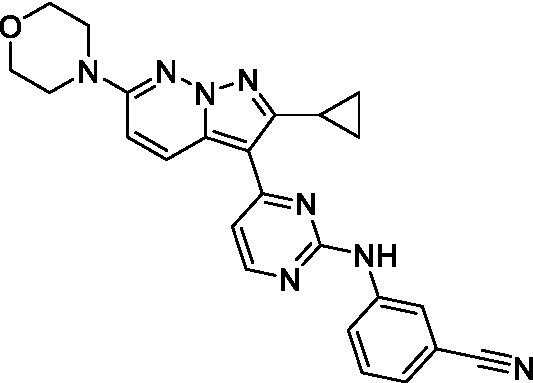	3.5 ± 0.7	> 50	22 ± 2.1
	**2d**	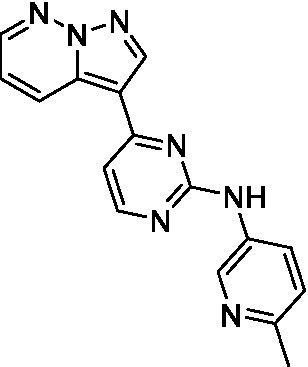	0.43 ± 0.13	> 50	< 0.62
	**2f**	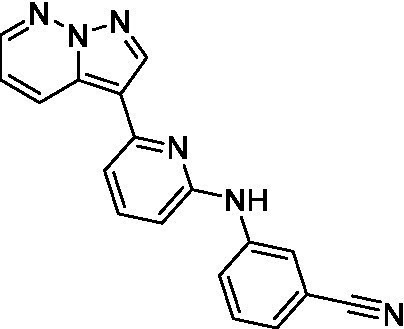	1.0 ± 0.01	> 50	> 50
	**2 h**	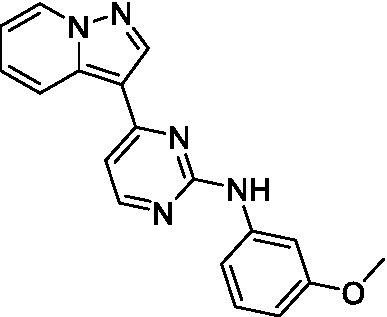	1.3 ± 0.26	> 50	4.2 ± 0.89
	**2i**	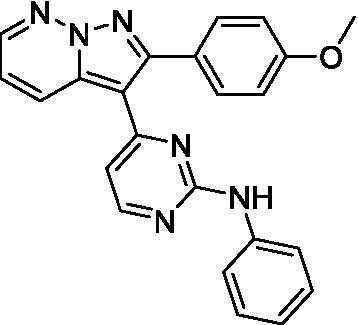	17 ± 3.5	> 50	48 ± 9.4
	**2j**	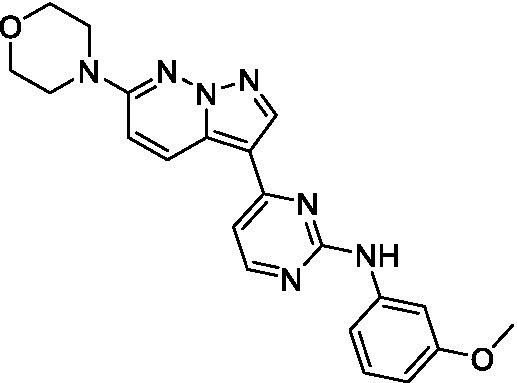	0.98 ± 0.13	> 50	4.1 ± 0.94
	**2 k**	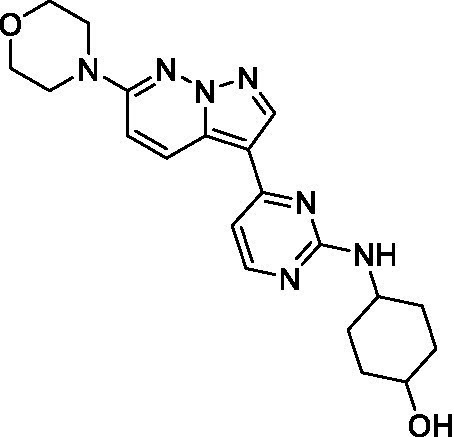	7.5 ± 2.5	> 50	9.9 ± 1.7
	**2 l**	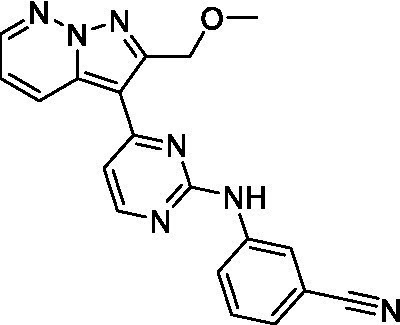	1.1 ± 0.16	> 50	14.2 ± 1.1
	**2 m**	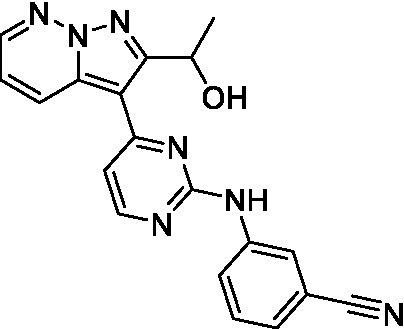	17 ± 0.76	> 50	*nt*
	**2n**	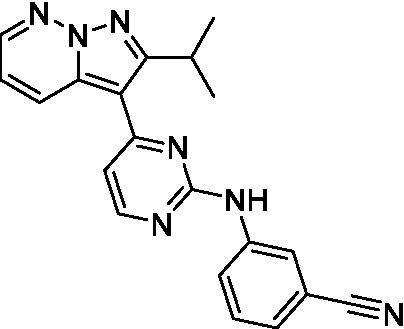	1.3 ± 0.33	33 ± 2.0	19 ± 1.1
	**2o**	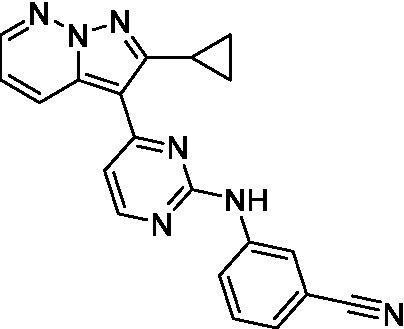	9.6 ± 6.6	> 50	14 ± 0.75
	**2p**	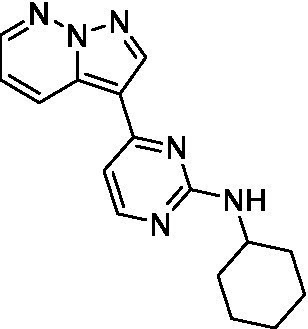	1.4 ± 0.76	> 50	5.5 ± 1.1
	**2q**	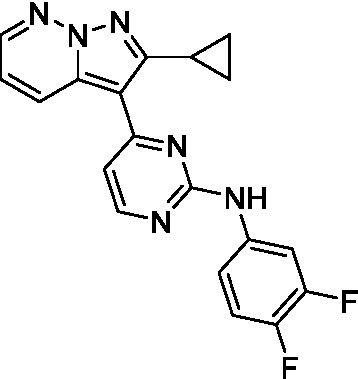	12 ± 7.8	> 50	50 ± 0
	**2r**	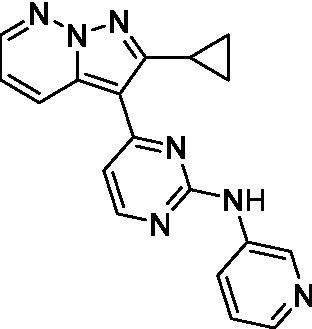	16 ± 3.7	> 50	31 ± 2.1
	**2 s**	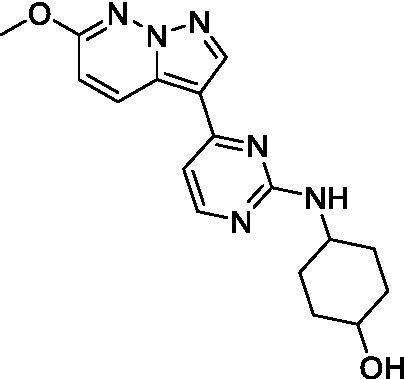	2.3 ± 1.0	> 50	30.5 ± 1.3
Imidazopyridines (Dichiara et al., 2022)	**3a**	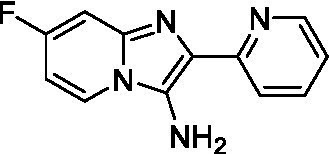	15 ± 4.7	*nt*	*nt*
	**3b**	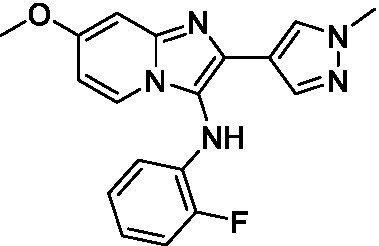	13 ± 6.7	*nt*	*nt*
	**3c**	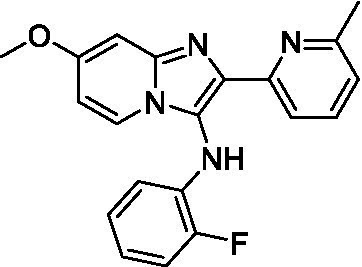	> 20	*nt*	*nt*
	**3d**	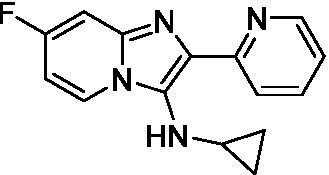	> 20	*nt*	*nt*
	**3e**	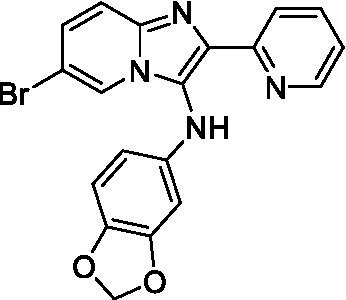	> 20	*nt*	*nt*
	**3f**	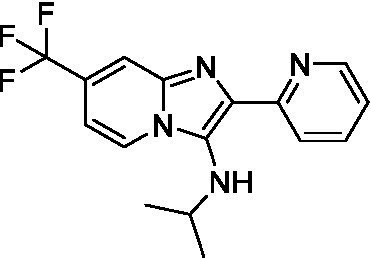	> 20	*nt*	*nt*
	**3 g**	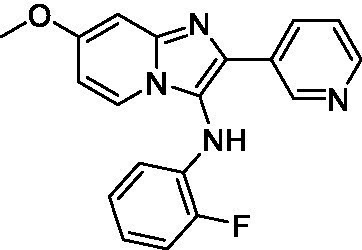	> 20	*nt*	*nt*
	**3 h**	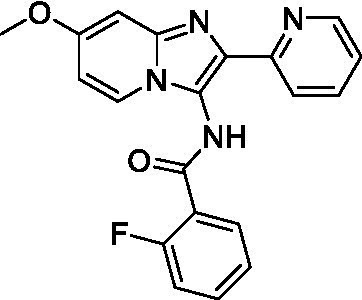	> 20	*nt*	*nt*
	**3i**	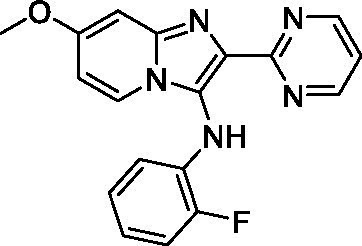	> 20	*nt*	*nt*
	**3j**	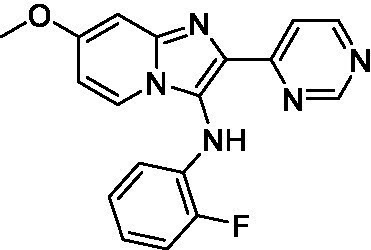	> 20	*nt*	*nt*
	**3 k**	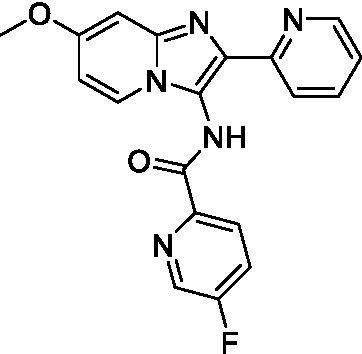	> 20	*nt*	*nt*
	**3 l**	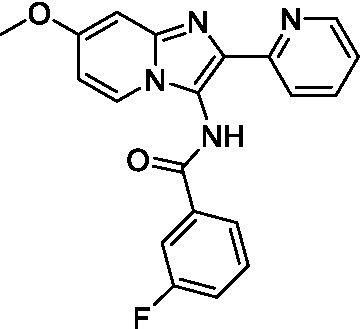	> 20	*nt*	*nt*
3-5-substituted azaindoles (Klug et al., 2021)	**4d**	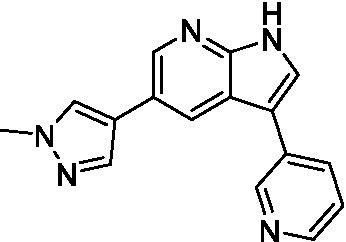	8.4 ± 0.71	> 50	17 ± 2.0
	**4e**	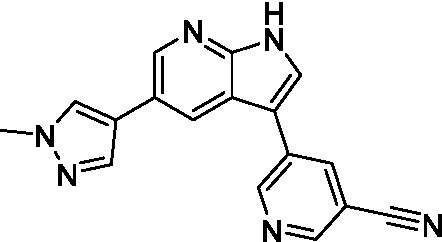	> 20	> 50	17 ± 2.0
	**4f**	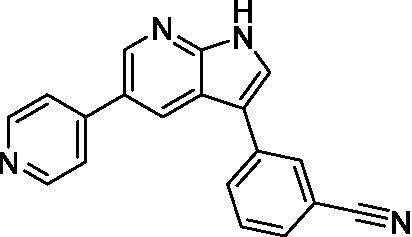	> 20	> 50	> 50
2,4-Disubstituted azaindoles	**5a**	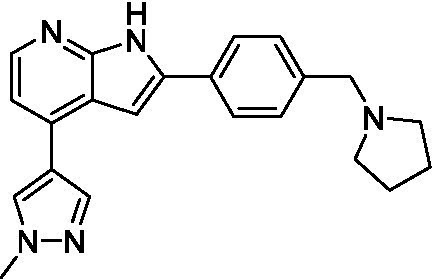	4.0 ± 0.36	16 ± 1.4	< 0.62
	**5b**	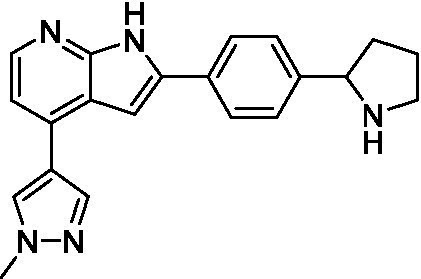	7.4 ± 0.25	5.7 ± 0.45	< 0.62
	**5c** (Diaz et al., 2014)	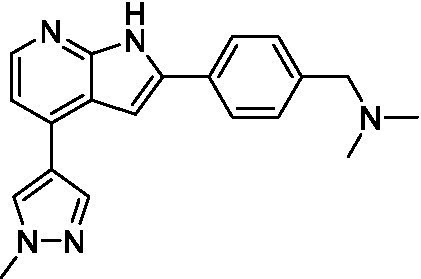	5.6 ± 0.29	11 ± 0.90	< 0.62
	**5d**	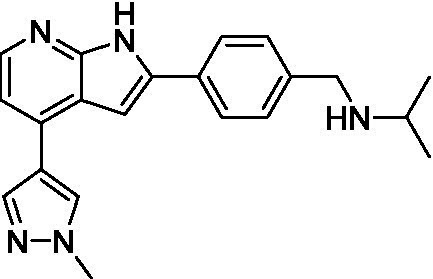	5.4 ± 0.21	1.3 ± 0.05	< 0.62
	**5e**	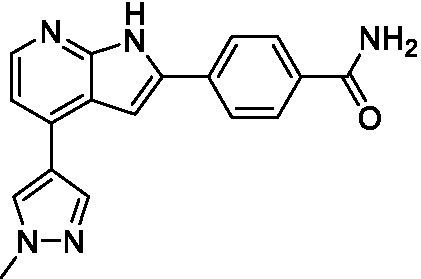	> 20	> 5.5 ± 0.00	*nt*
Aminopurines (Singh et al., 2020)	**6a** (Diaz et al., 2014)	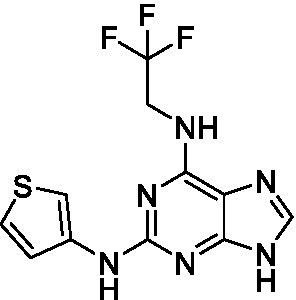	11 ± 0.60	50 ± 0.00	*nt*
	**6b**	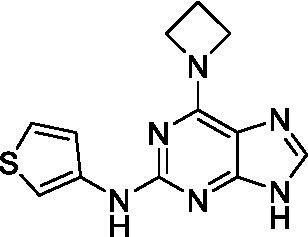	14 ± 5.7	> 50	*nt*
	**6c**	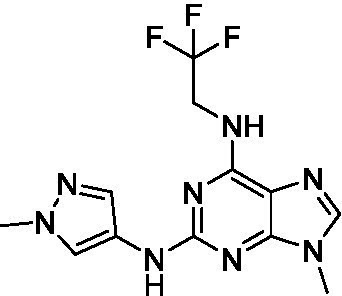	> 20	> 50	> 50
	**6d**	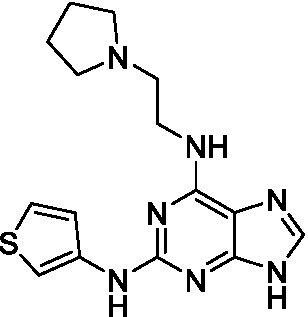	> 20	> 50	> 50
	**6e**	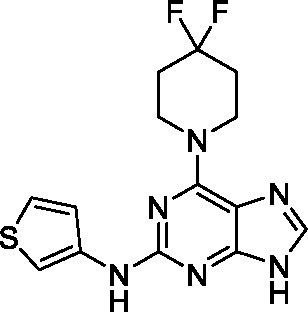	> 20	> 50	> 50
4-Aminoquinolines (Mehta et al., 2018)	**7a**	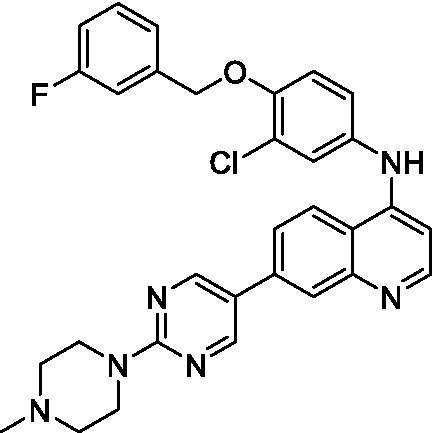	16 ± 4.1	*nt*	*nt*
Carbazoles (Singh et al., 2023)	**8a**	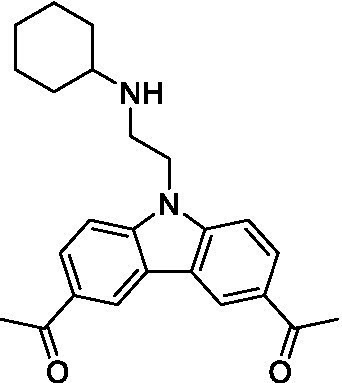	5.1 ± 0.06	*nt*	*nt*
	**8b**	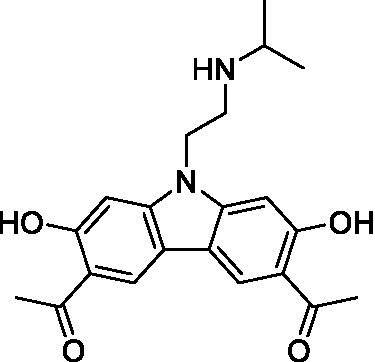	8.8 ± 0.71	*nt*	*nt*
	**8c**	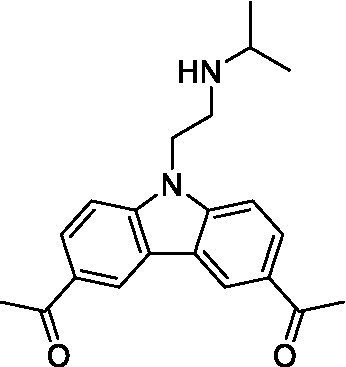	4.8 ± 0.09	*nt*	*nt*
	**8d**	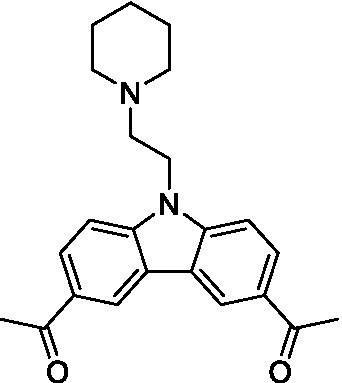	4.3 ± 0.22	*nt*	*nt*
	**8e**	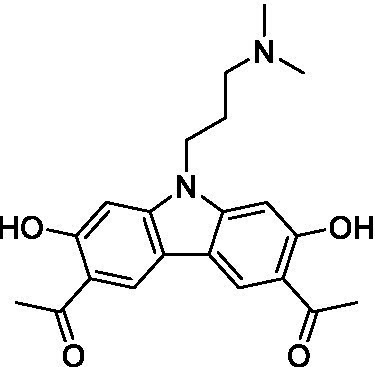	17 ± 0.65	*nt*	*nt*
	**8f**	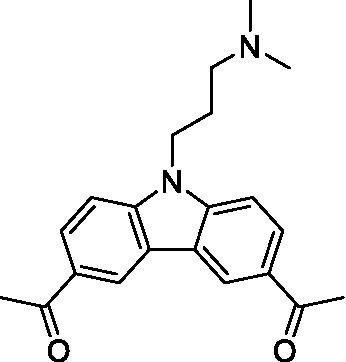	17 ± 0.2	*nt*	*nt*
	**8 g**	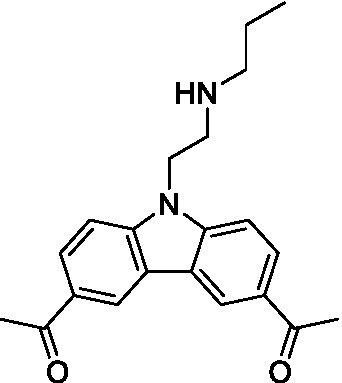	9.6 ± 0.05	*nt*	*nt*
	**8 h**	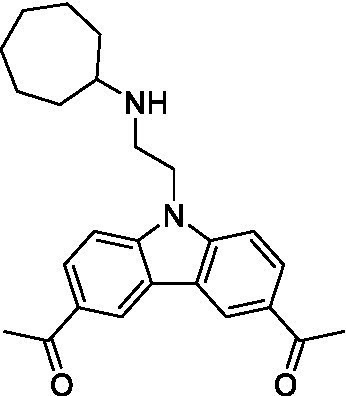	13 ± 2.6	*nt*	*nt*
	**8i**	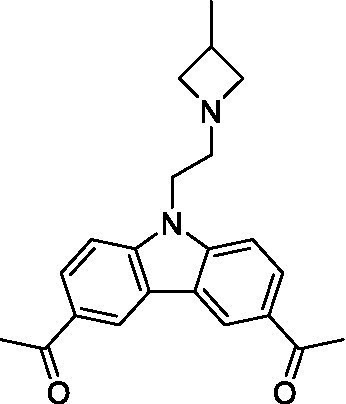	18 ± 0.45	*nt*	*nt*
	**8j**	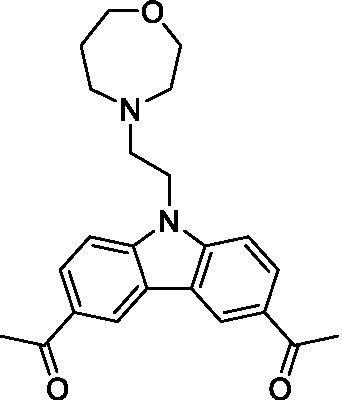	10 ± 1.4	*nt*	*nt*
	**8 k**	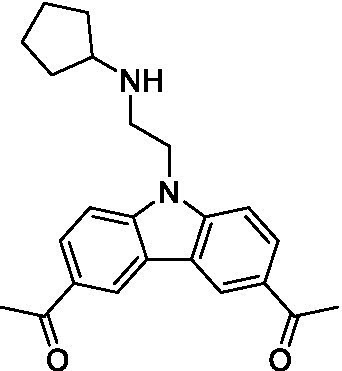	9.0 ± 0.01	*nt*	*nt*
	**8 l**	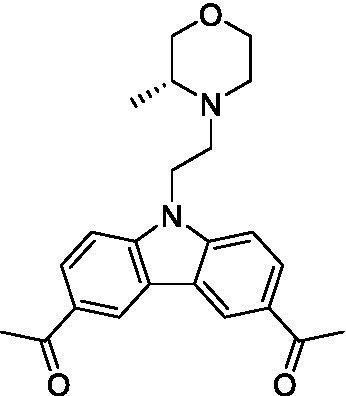	16 ± 2.6	*nt*	*nt*
Reference Drugs	Azithromycin		0.02 ± 0.01		
	Chlorhexidine		5.8 ± 0.22		
	Pentamidine		> 50		

nt, not tested.

**Table 3:** Active compounds against *Balamuthia mandrillaris*, *n* = 3.

Class	ID	Structure	*Balamuthia mandrillaris*	MRC5 CC_50_	L6 CC_50_
			EC_50_ (μM) ± SD	(μM) ± SD	(μM) ± SD
Benzoxazepinoindazoles (Klug et al., 2019)	**1a**	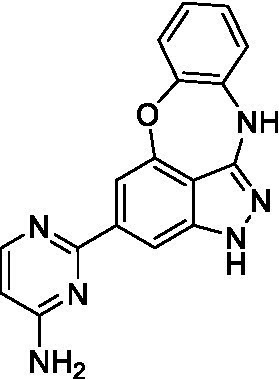	17 ± 3.0	> 50	32 ± 2.5
	**1b**	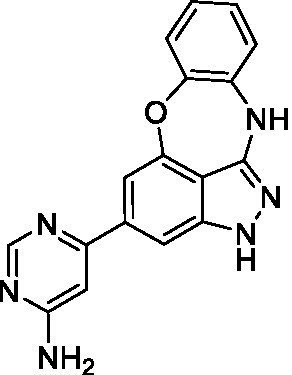	1.8 ± 0.46	> 50	1.0 ± 0.14
	**1c**	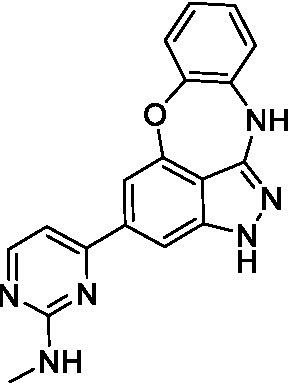	1.9 ± 1.3	> 50	6.3 ± 0.55
	**1d**	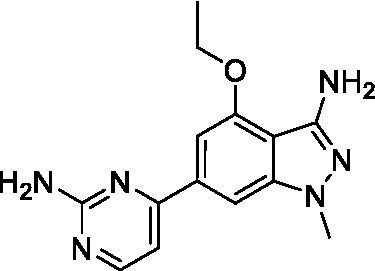	9.5 ± 0.23	> 50	> 17 ± 0.05
	**1e**	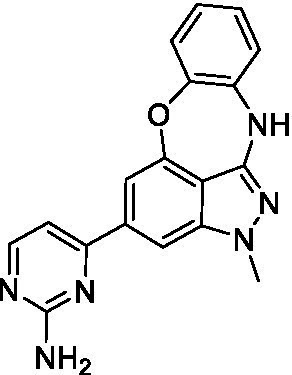	14 ± 0.29	> 50	> 50
Pyrazolopyridazines (Tear et al., 2020)	**2a**	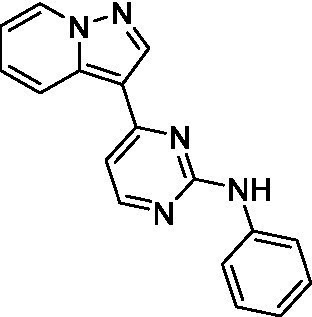	4.2 ± 0.6	> 50	3.2 ± 0.08
	**2b**	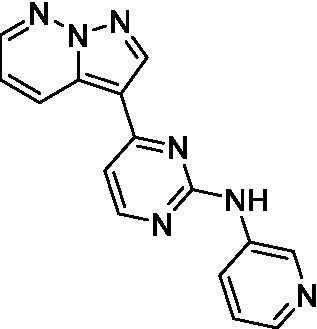	5.0 ± 0.1	> 50	< 0.62
	**2d**	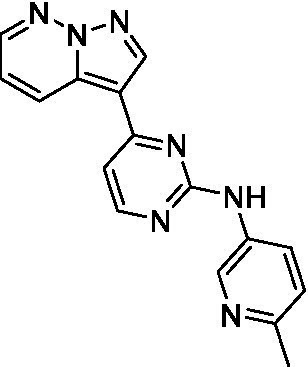	1.6 ± 0.1	> 50	< 0.62
	**2f**	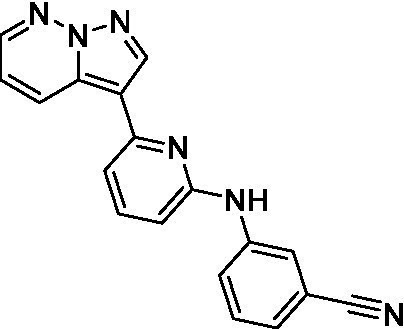	> 20	> 50	> 50
	**2 g**	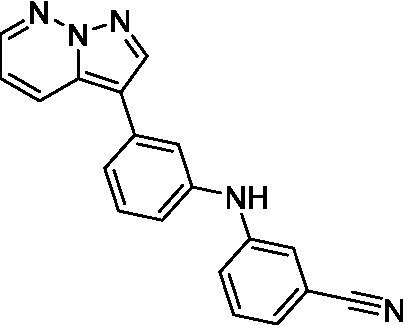	> 20	19 ± 1.0	28 ± 1.1
	**2 h**	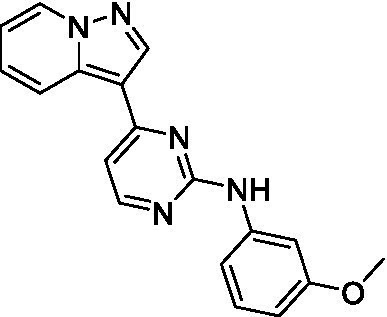	3.3 ± 0.97	> 50	*nt*
	**2j**	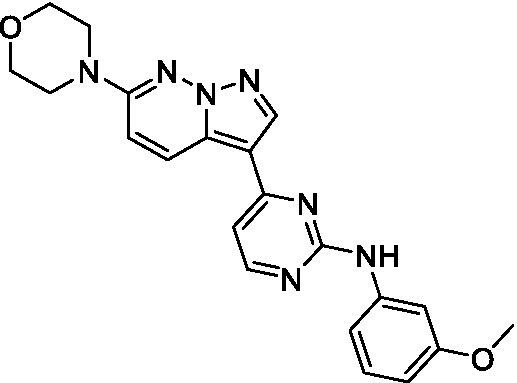	4.9 ± 1.6	> 50	4.1 ± 0.94
	**2 k**	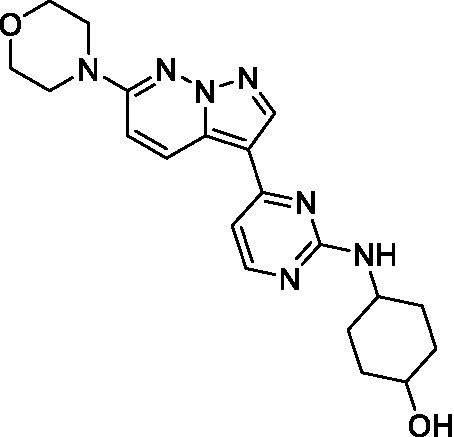	> 20	> 50	9.9 ± 1.7
	**2p**	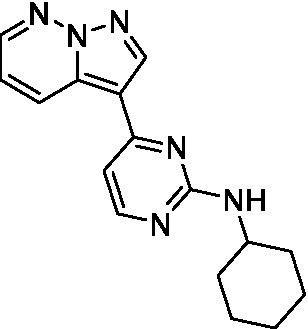	7.5 ± 3.1	> 50	5.5 ± 1.1
	**2r**	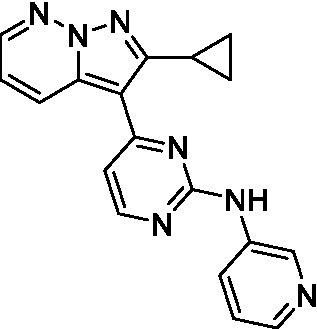	13 ± 7.5	> 50	31 ± 2.1
	**2 s**	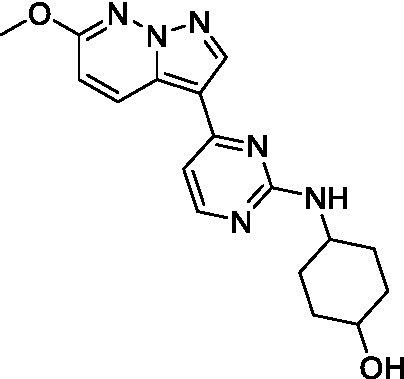	11 ± 4.2	> 50	31 ± 1.3
	**2 t**	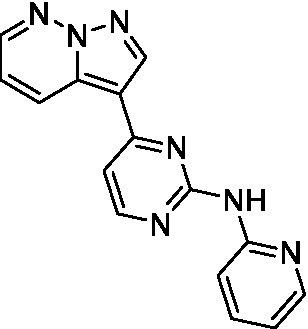	10 ± 2.8	> 50	3.7 ± 0.46
	**2u**	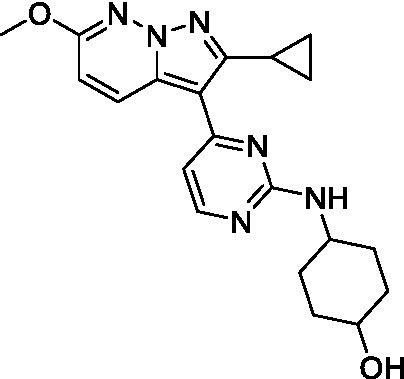	9.4 ± 3.4	> 50	> 50
3-5-substituted azaindoles (Klug et al., 2021)	**4a**	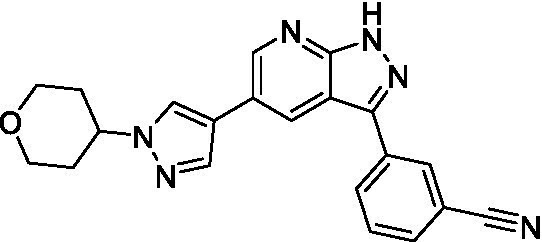	3.3 ± 0.62	> 50	2.8 ± 0.92
	**4b**	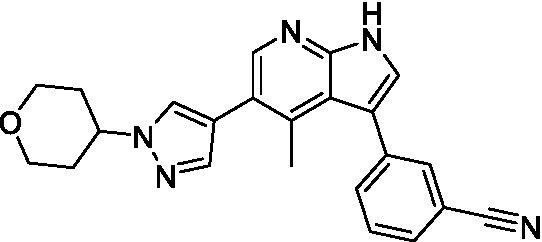	1.0 ± 0.12	> 50	11 ± 1.5
	**4e**	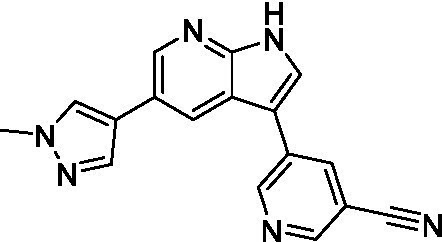	8.3 ± 1.3	> 50	17 ± 2.0
2,4-substituted azaindoles	**5b**	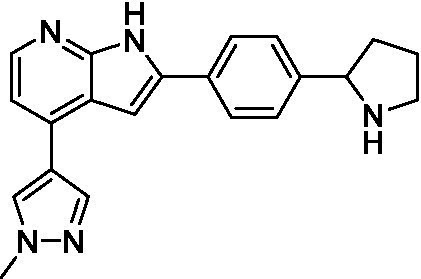	7.25 ± 0.7	5.7 ± 0.45	< 0.62
	**5c** (Diaz et al., 2014)	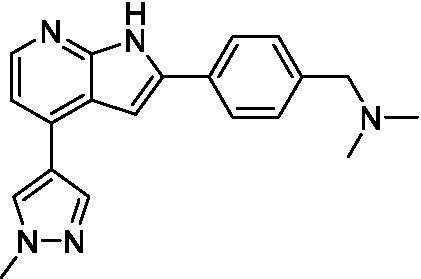	12 ± 0.09	11 ± 0.90	< 0.62
	**5e**	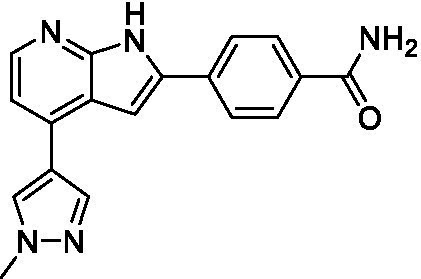	1.8 ± 0.24	> 5.5 ± 0.00	*nt*
4-Aminoquinolines (Bachovchin et al., 2019)	**7b**	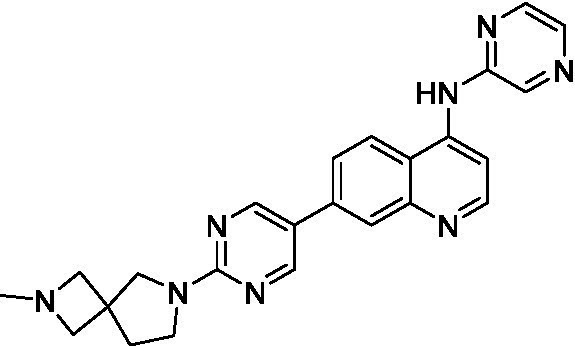	1.4 ± 0.17	*nt*	*nt*
Carbazoles (Singh et al., 2023)	**8a**	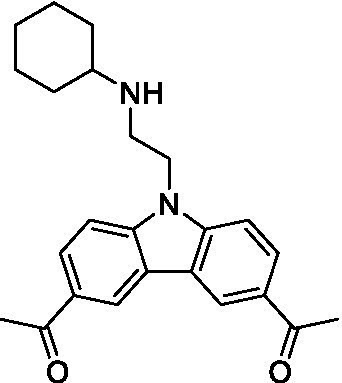	6.8 ± 0.1	*nt*	*nt*
	**8b**	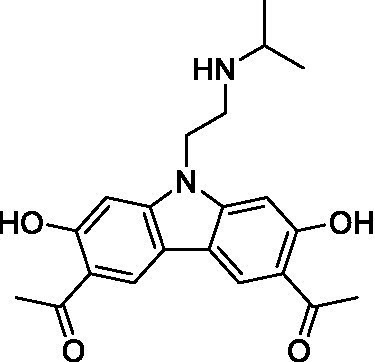	11 ± 0.35	*nt*	*nt*
	**8c**	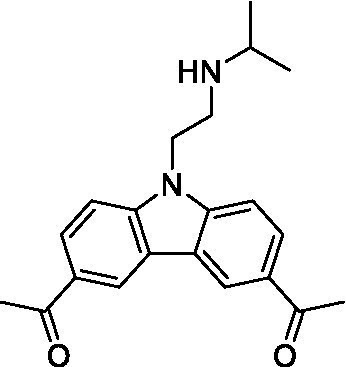	2.4 ± 0.17	*nt*	*nt*
	**8d**	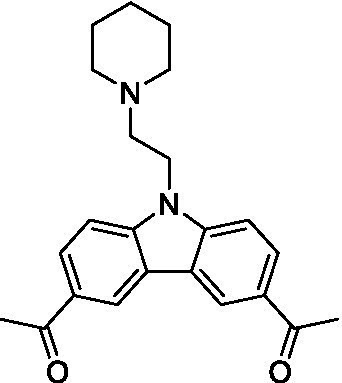	12 ± 0.26	*nt*	*nt*
	**8f**	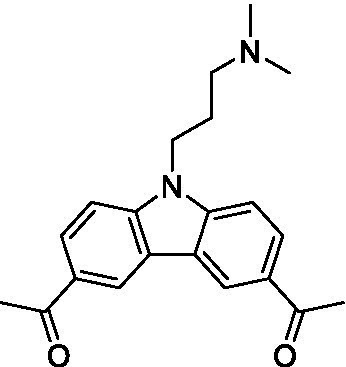	19 ± 1.1	*nt*	*nt*
	**8 g**	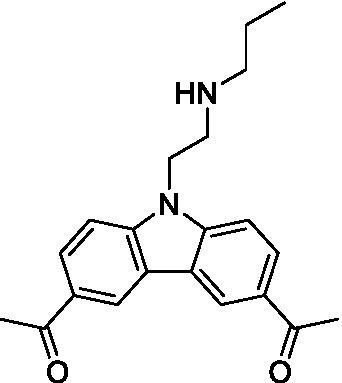	12 ± 0.07	*nt*	*nt*
	**8 h**	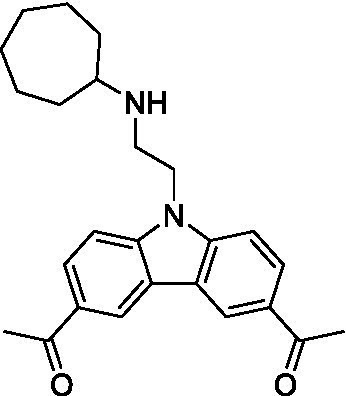	7.3 ± 0.38	*nt*	*nt*
	**8i**	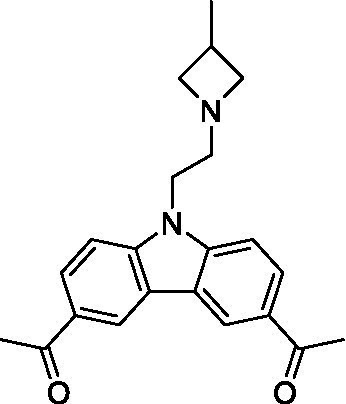	7.3 ± 0.28	*nt*	*nt*
	**8j**	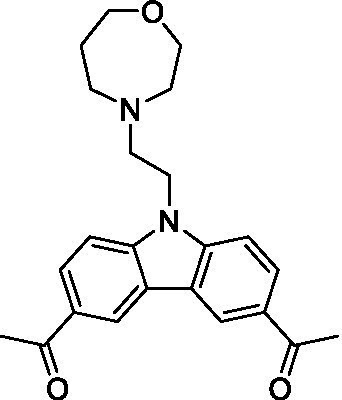	3.3 ± 0.24	*nt*	*nt*
	**8 k**	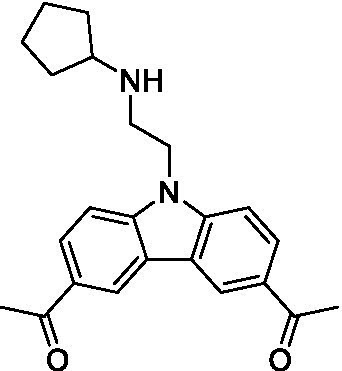	7.4 ± 0.36	*nt*	*nt*
	**8 l**	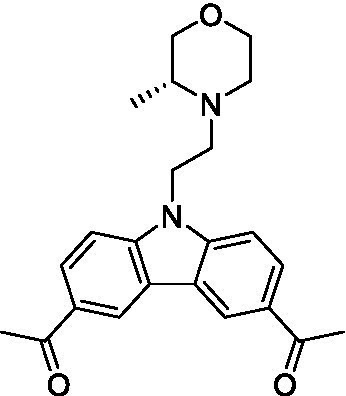	13 ± 0.35	*nt*	*nt*
Reference Drugs	Azithromycin		>20		
	Chlorhexidine		1.6 ± 0.17		
	Pentamidine		18.35 ± 1.47		

nt, not tested.

## Conclusion

This work represents a significant screening campaign against three pFLA from which we have identified several new chemical series with sub-to-low-micromolar potency. Many of the compounds discovered and described here have better potency and selectivity profiles than the suggested multi-drug cocktail treatment regimen against *A. castellanii*, *N. fowleri*, or *B. mandrillaris* from the U.S. Centers for Disease Control and Prevention (CDC). These pharmacophores are currently being further optimized using medicinal chemistry to develop compounds with greater potency for these pFLA, selectivity, and higher brain exposure. The results of these studies will be reported in due course.

## Data availability statement

The datasets presented in this study can be found in online repositories. The names of the repository/repositories and accession number(s) can be found in the article/Supplementary material.

## Author contributions

CR, MB, and LF: conceptualization. LF, MB, MK, HE, SE, CLu, DKl, BS, AQ, KB, WT, AS, KF, SB, MR, CLe, EB, JA, RD-G, GC-P, GP-M, CB-N, CG-L, DG-P, MN, and CR: methodology. CR, DKy, MP, and LF: validation and supervision. LF and CR: formal analysis and writing – original draft preparation. DKy, MP, and LF: resources and funding acquisition. LF, MB, DKl, BS, KB, WT, SB, MK, HE, SE, CLu, CB-N, and CR: data curation. LF, MB, MK, HE, SE, CLu, DKl, BS, AQ, KB, WT, AS, KF, SB, MR, CLe, EB, JA, RD-G, GC-P, RG-H, GP-M, CB-N, CG-L, LR-P, FG, DG-P, MN, KM-W, MP, DKy, and CR: writing – review and editing. All authors contributed to the article and approved the submitted version

## Funding

This study was supported by the Georgia Research Alliance (DKy). Additional funding was received from the National Institute of Allergy and Infectious Diseases (MP and MN, R01AI114685; MP, 1R21AI127594 and R01AI124046; and CC, R21AI126296; https://www.niaid.nih.gov/), the Spanish Ministerio de Economí a, Industria y Competitividad (MN, SAF2015-71444-P; DG-P, SAF2016-79957-R; http://www.mineco.gob.es), Subdireccion General de Redes ´ y Centros de Investigacion Cooperativa-Red de Investigación Cooperativa en Enfermedades Tropicales [RICET, https://www.ricet.es/; M.N., RD16/0027/0019; DG-P, the MCIN/AEI/10.13039/501100011033 (PID2019-109623RB-I00), the MCIN/AEI/10.13039/501100011033, and FEDER Una manera de hacer Europa (2016-79957-R)], and RTI2018-097210-B-I00 (MINCIU-FEDER) to FG. An ACS MEDI Predoctoral Fellowship for DKl is gratefully acknowledged, as is support from the National Science Foundation for KF (CHE-1262734). This project has been co-funded by the Tres Cantos Open Lab Foundation projects TC-007 and TC-164.

## Conflict of interest

The authors declare that the research was conducted in the absence of any commercial or financial relationships that could be construed as a potential conflict of interest.

## Publisher’s note

All claims expressed in this article are solely those of the authors and do not necessarily represent those of their affiliated organizations, or those of the publisher, the editors and the reviewers. Any product that may be evaluated in this article, or claim that may be made by its manufacturer, is not guaranteed or endorsed by the publisher.
