# Corrigendum: Identification of novel anti-amoebic pharmacophores from kinase inhibitor chemotypes

**DOI:** 10.3389/fmicb.2023.1304196

**Published:** 2023-10-25

**Authors:** Lori Ferrins, Melissa J. Buskes, Madison M. Kapteyn, Hannah N. Engels, Suzanne E. Enos, Chenyang Lu, Dana M. Klug, Baljinder Singh, Antonio Quotadamo, Kelly Bachovchin, Westley F. Tear, Andrew E. Spaulding, Katherine C. Forbes, Seema Bag, Mitch Rivers, Catherine LeBlanc, Erin Burchfield, Jeremy R. Armand, Rosario Diaz-Gonzalez, Gloria Ceballos-Perez, Raquel García-Hernández, Guiomar Pérez-Moreno, Cristina Bosch-Navarrete, Claudia Gómez-Liñán, Luis Miguel Ruiz-Pérez, Francisco Gamarro, Dolores González-Pacanowska, Miguel Navarro, Kojo Mensa-Wilmot, Michael P. Pollastri, Dennis E. Kyle, Christopher A. Rice

**Affiliations:** ^1^Department of Chemistry and Chemical Biology, Northeastern University, Boston, MA, United States; ^2^Center for Tropical and Emerging Global Diseases, University of Georgia, Athens, GA, United States; ^3^Department of Pharmaceutical and Biomedical Sciences, College of Pharmacy, University of Georgia, Athens, GA, United States; ^4^Department of Comparative Pathobiology, College of Veterinary Medicine, Purdue University, West Lafayette, IN, United States; ^5^Clinical and Experimental Medicine PhD Program, University of Modena and Reggio Emilia, Modena, Italy; ^6^Instituto de Parasitología y Biomedicina “López-Neyra” Consejo Superior de Investigaciones Científicas (CSIC), Granada, Spain; ^7^Department of Molecular and Cellular Biology, Kennesaw State University, Kennesaw, GA, United States

**Keywords:** pathogenic free-living amoeba, *Acanthamoeba* species, *Naegleria fowleri*, *Balamuthia mandrillaris*, kinase inhibitors, cross-screening, hit-identification

In the published article, there was an error in the author list, and author “Claudia Gómez-Liñán” was erroneously excluded. The corrected author list appears below.

Lori Ferrins^1*^, Melissa J. Buskes^1^, Madison M. Kapteyn^2^, Hannah N. Engels^2^, Suzanne E. Enos^2,3^, Chenyang Lu^4^, Dana M. Klug^1^, Baljinder Singh^1^, Antonio Quotadamo^1,5^, Kelly Bachovchin^1^, Westley F. Tear^1^, Andrew E. Spaulding^1^, Katherine C. Forbes^1^, Seema Bag^1^, Mitch Rivers^1^, Catherine LeBlanc^1^, Erin Burchfield^1^, Jeremy R. Armand^1^, Rosario Diaz-Gonzalez^6^, Gloria Ceballos-Perez^6^, Raquel García-Hernández^6^, Guiomar Pérez-Moreno^6^, Cristina Bosch-Navarrete^6^, Claudia Gómez-Liñán^6^, Luis Miguel Ruiz-Pérez^6^, Francisco Gamarro^6^, Dolores González-Pacanowska^6^, Miguel Navarro^6^, Kojo Mensa-Wilmot^7^, Michael P. Pollastri^1^, Dennis E. Kyle^2^ and Christopher A. Rice^2,3,4^^*^^†^

In the original article there was also an error in the **Author Contribution** section.

The correct author contributions section appears below:

## Author contributions

CR, MB, and LF: conceptualization. LF, MB, MK, HE, SE, CLu, DKl, BS, AQ, KB, WT, AS, KF, SB, MR, CLe, EB, JA, RD-G, GC-P, GP-M, CB-N, CG-L, DG-P, MN, and CR: methodology. CR, DKy, MP, and LF: validation and supervision. LF and CR: formal analysis and writing—original draft preparation. DKy, MP, and LF: resources and funding acquisition. LF, MB, DKl, BS, KB, WT, SB, MK, HE, SE, CLu, CB-N, and CR: data curation. LF, MB, MK, HE, SE, CLu, DKl, BS, AQ, KB, WT, AS, KF, SB, MR, CLe, EB, JA, RD-G, GC-P, RG-H, GP-M, CB-N, CG-L, LR-P, FG, DG-P, MN, KM-W, MP, DKy, and CR: writing—review and editing. All authors contributed to the article and approved the submitted version.

The authors apologize for this error and state that this does not change the scientific conclusions of the article in any way. The original article has been updated.

