# Supplementary material for: Identification of novel anti-amoebic pharmacophores from kinase inhibitor chemotypes
Source: Front Microbiol. 2023 May 10;14:1149145. doi: 10.3389/fmicb.2023.1149145 (PMC10206040; doi:10.3389/fmicb.2023.1149145)
Supplement: Supplementary file 1 [file Table_1.DOCX]

Lori Ferrins,^1^* Melissa J. Buskes,^1^ Madison M. Kapteyn,^2^ Hannah N. Engels,^2^ Suzanne E. Enos,^2,3^ Chenyang Lu,^4^ Dana M. Klug,^1^ Baljinder Singh,^1^ Antonio Quotadamo,^1,5^ Kelly Bachovchin,^1^ Westley F. Tear,^1^ Andrew E. Spaulding,^1^ Katherine C. Forbes,^1^ Seema Bag,^1^ Mitch Rivers,^1^ Catherine LeBlanc,^1^ Erin Burchfield,^1^ Jeremy R. Armand,^1^ Rosario Diaz-Gonzalez,^6^ Gloria Ceballos-Perez,^6^ Raquel García-Hernández,^6^ Guiomar Pérez-Moreno,^6^ Luis Miguel Ruiz-Pérez,^6^ Francisco Gamarro,^6^ Dolores González-Pacanowska,^6^ Miguel Navarro,^6^ Kojo Mensa-Wilmot,^7^ Michael P. Pollastri,^1^ Dennis E. Kyle,^2^ Christopher A. Rice,^2,3,4†Δ^*

1. Department of Chemistry and Chemical Biology, Northeastern University, Boston, Massachusetts, 02115, United States of America.

2. Center for Tropical and Emerging Global Diseases, University of Georgia, Athens, Georgia, 30602, United States of America.

3. Department of Pharmaceutical and Biomedical Sciences, College of Pharmacy, University of Georgia, Athens, Georgia, 30602, United States of America.

4. Department of Comparative Pathobiology, College of Veterinary Medicine, Purdue University, West Lafayette, Indiana, 47907, United States of America.

5. Clinical and Experimental Medicine PhD Program, University of Modena and Reggio Emilia, 41121 Modena, Italy.

6. Instituto de Parasitología y Biomedicina "López-Neyra" Consejo Superior de Investigaciones Científicas (CSIC), Granada 18100, Spain.

7. Department of Molecular and Cellular Biology, Kennesaw State University, Kennesaw, Georgia, 30144, United States of America

† Current address: Purdue Institute for Drug Discovery (PIDD), Purdue University, West Lafayette, Indiana, 47907, United States of America.

Δ Current address: Purdue Institute of Inflammation, Immunology and Infectious Disease (PI4D), Purdue University, West Lafayette, Indiana, 47907, United States of America.

* Corresponding authors: LF Email: [l.ferrins@northeastern.edu](mailto:l.ferrins@northeastern.edu). Tel: +1-(617)-373-3715; CAR Email: [carice@purdue.edu](mailto:carice@purdue.edu). Tel: +1-(765)-494-0005.

Table of Contents

[**Biological Protocols** 3](#_Toc123749720)

[*Cytotoxicity assay in THP-1.* 3](#_Toc123749721)

[**Chemistry Experimental for compounds not previously published** 3](#_Toc123749722)

[Scheme 1. Synthesis of diarylimidazole derivatives. 3](#_Toc123749723)

[Scheme 2. Synthesis of diarylimidazole amide derivatives. 3](#_Toc123749724)

[Scheme 3. Synthesis of 4,5-dimethyl-1*H*-pyrazol-3-amine derivatives. 8](#_Toc123749725)

[Scheme 4. Synthesis of 4,5-dimethyl-1*H*-pyrazol-3-amine derivatives. 11](#_Toc123749726)

[Scheme 5. Synthesis of 4,5-dimethyl-1*H*-pyrazol-3-amine derivatives. 14](#_Toc123749727)

[Scheme 6. Synthesis of the aminopyrimidine derivative. 16](#_Toc123749728)

[**Table S1.** Additional compounds not presented in the manuscript. 17](#_Toc123749729)

## Table S2. Additional cytotoxicity data for all tested compounds…………………………………………20

[**Figure S1.** Lipophilic ligand efficiency of all active compounds against *Acanthamoeba castellanii*, *Naegleria fowleri*, and *Balamuthia mandrillaris.* LLE calculated as pEC_50_-LogD_7.4_. 24](#_Toc123749730)

[**References** 25](#_Toc123749731)

**Biological Protocols**

*Cytotoxicity assay in THP-1.* Cellular toxicity of all compounds was determined using the colorimetric MTT-based assay after incubation at 37 °C for 72 h in the presence of increasing concentrations of compounds (final maximal concentration was 50 μM in 0.5% DMSO per well) [1]. The results are expressed as EC_50_ values, the concentration of compound that reduces cell growth by 50% versus untreated control cells. Assays were performed in duplicate at least twice to achieve a minimal n=3 per dose response.

# **Chemistry Experimental for compounds not previously published**

**General Chemistry.** All starting materials were commercially procured and were used without further purification, unless specified. Reaction solvents were purified by passage through alumina columns on a purification system manufactured by Innovative Technology (Newburyport, MA). NMR spectra were obtained on Varian NMR systems, operating at 400 or 500 MHz for 1 H acquisitions. LCMS analysis was performed using a Waters Alliance reverse phase HPLC (columns Waters SunFire C18 4.6 × 50 mm, 3.5 μm, or Waters SunFire C8 4.6 × 50 mm, 3.5 μm), with single-wavelength UV−visible detector and LCT Premier time-of-flight mass spectrometer (electrospray ionization) or Waters Micromass ZQ detector (electrospray ionization). Optical rotations were obtained on a Jasco P-2000. Where required, final compounds were purified by preparative reverse phase HPLC (columns Waters Symmetry RP8 30 × 50 mm, 5 μm column, or OBD RP18 30 × 50 mm, 5 μm), with a single wavelength UV−visible detector and Waters Micromass ZQ (electrospray ionization). All final compounds have purities greater than 95% based upon LC/MS analysis.

## Scheme 1. Synthesis of diarylimidazole derivatives.

*^a^Reagents and reaction conditions:* (i) NaOH (50%, v/v), TEAC, CH_3_Cl, CH_2_Cl_2_, 40 °C, 3–8 h; (ii) appropriately substituted pyridin-2-amine, aldehyde, Yb(OTf)_3_, 120 °C, microwave, 40 min.

## Scheme 2. Synthesis of diarylimidazole amide derivatives.

*^a^Reagents and reaction conditions:* (i) 3M aqueous HBr, 110 °C, 3h; (ii) carboxylic acid derivative, HATU, DIEA, DMF, 18 h.

**General Procedure A: (for the synthesis of Isocyanides)**

To the vigorously stirred solution of a 50% sodium hydroxide solution (12 mL) was added TEBA chloride (0.125 g), The reaction mixture was efficiently stirred and heated at 40°C and a solution of the selected amine (1 eq.), chloroform (1.05 eq.) in DCM (12 mL) was added dropwise in 50’ period. The reaction mixture was efficiently stirred and heated at 40◦C (gently refluxing) for 3–8 h. The progress of the reaction was thoroughly monitored by means of TLC. The reaction mixture was allowed to cool to r.t., and cold water (50–100 mL) was added. The aqueous phase was extracted with methylene chloride. The organic layer was washed with concentrated brine solution and dried over sodium sulphate. The drying agent was filtered off, and the solvent was evaporated under reduced pressure. The residue was subjected to the next step without purification

**General Procedure B: (amide coupling)**

HATU (1.1 eq.) carboxylic acid derivative (1.0 eq.), amine derivative (1.0 eq.), DIEA (3 eq) in DMF (1.5 mL) at RT overnight. The reaction was quenched with water 5 ml and after 10 h in fridge the ppt obtained was collected and dried to give the desiderate compound.

*7-Fluoro-2-(pyridin-2-yl)imidazo[1,2-a]pyridin-3-amine* (**3a**)

The title compound was isolated from the reaction to produce **3d**. It was isolated as a dark orange oil (18.6 mg, 16%). LCMS [M+H]^+^ 229.0 m/z; ^1^H NMR (500 MHz, Chloroform-*d*) δ 8.60 – 8.48 (m, 1H), 8.10 (d, *J* = 8.1 Hz, 1H), 7.80 – 7.64 (m, 2H), 7.13 (dd, *J* = 9.8, 2.4 Hz, 1H), 7.10 (ddd, *J* = 7.5, 4.9, 1.2 Hz, 1H), 6.66 (td, *J* = 7.3, 2.4 Hz, 1H), 5.31 (s, 2H).

*N-(2-Fluorophenyl)-7-methoxy-2-(1-methyl-1H-pyrazol-4-yl)imidazo[1,2-a]pyridin-3-amine* (**3b**)

The title compound was prepared according to General Procedure A utilizing 0.402 mmol of 4-methoxypyridin-2-amine. The material was purified by reverse phase column chromatography, eluting with 30-70% methanol in water to give the title compound as a light orange solid (35 mg, 26%). LCMS [M+H]^+^ 338.0 m/z; ^1^H NMR (500 MHz, Chloroform-*d*) δ 7.85 (s, 1H), 7.78 (s, 1H), 7.68 (d, *J* = 7.4 Hz, 1H), 7.13 (ddd, *J* = 11.6, 8.1, 1.5 Hz, 1H), 6.89 (d, *J* = 2.3 Hz, 1H), 6.87 – 6.82 (m, 1H), 6.80 – 6.73 (m, 1H), 6.51 (dd, *J* = 7.4, 2.4 Hz, 1H), 6.28 (ddd, *J* = 9.2, 8.0, 1.6 Hz, 1H), 5.70 (s, 1H), 3.89 (s, 3H), 3.87 (s, 3H).

*N-(2-Fluorophenyl)-7-methoxy-2-(6-methylpyridin-2-yl)imidazo[1,2-a]pyridin-3-amine* (**3c**)

The title compound was prepared according to General Procedure A utilizing 0.402 mmol of 4-methoxypyridin-2-amine. The material was purified by reverse phase column chromatography, eluting with 30-70% methanol in water to give the title compound as a light brown solid (40 mg, 28%). LCMS [M+H]^+^ 349.1 m/z; ^1^H NMR (500 MHz, Chloroform-*d*) δ 8.26 (s, 1H), 7.91 (d, *J* = 7.8 Hz, 1H), 7.66 – 7.57 (m, 2H), 7.12 (dd, *J* = 11.3, 8.1 Hz, 1H), 6.98 (d, *J* = 7.6 Hz, 1H), 6.94 (s, 1H), 6.88 (t, *J* = 7.7 Hz, 1H), 6.81 (q, *J* = 6.9 Hz, 1H), 6.60 – 6.49 (m, 1H), 6.21 (t, *J* = 8.3 Hz, 1H), 3.89 (s, 3H), 2.54 (s, 3H).

*N-Cyclopropyl-7-fluoro-2-(pyridin-2-yl)imidazo[1,2-a]pyridin-3-amine* (**3d**)

The title compound was prepared according to General Procedure B utilizing 0.446 mmol of 4-fluoropyridin-2-amine. The material was purified by reverse phase column chromatography, eluting with 30-70% methanol in water to give the title compound as a dark orange oil (40.6 mg, 34%). LCMS [M+H]^+^ 269.1 m/z; ^1^H NMR (500 MHz, Chloroform-*d*) δ 8.52 (dt, *J* = 4.8, 1.5 Hz, 1H), 8.29 (dd, *J* = 7.6, 5.8 Hz, 1H), 8.11 (dt, *J* = 8.0, 1.1 Hz, 1H), 7.73 (td, *J* = 7.8, 1.8 Hz, 1H), 7.18 – 7.08 (m, 2H), 6.91 (s, 1H), 6.64 (td, *J* = 7.4, 2.5 Hz, 1H), 2.74 – 2.63 (m, 1H), 0.70 (t, *J* = 1.7 Hz, 1H), 0.68 (p, *J* = 2.2, 1.7 Hz, 3H).

*N-(Propan-2-yl)-2-(pyridin-2-yl)-7-(trifluoromethyl)imidazo[1,2-a]pyridin-3-amine* (**3f**)

The title compound was prepared according to General Procedure A utilizing 0.308 mmol of 4-(trifluoromethyl)pyridin-2-amine. The material was purified by reverse phase column chromatography, eluting with 30-70% methanol in water to give the title compound as a yellow solid (95 mg, 97%). LCMS [M+H]^+^ 321.1 m/z; ^1^H NMR (500 MHz, Chloroform-*d*) δ 8.57 (ddd, *J* = 4.9, 1.9, 1.0 Hz, 1H), 8.20 (dt, *J* = 8.1, 1.1 Hz, 1H), 8.08 – 8.02 (m, 1H), 7.84 (dt, *J* = 2.1, 1.0 Hz, 1H), 7.78 (td, *J* = 7.7, 1.8 Hz, 1H), 7.18 (ddd, *J* = 7.5, 4.9, 1.2 Hz, 1H), 6.91 (dd, *J* = 7.2, 1.7 Hz, 1H), 6.19 (d, *J* = 9.9 Hz, 1H), 3.49 (dp, *J* = 10.0, 6.4 Hz, 1H), 1.19 (d, *J* = 6.4 Hz, 6H).

*N-(2-fluorophenyl)-7-methoxy-2-(pyridin-3-yl)imidazo[1,2-a]pyridin-3-amine* (**3g**)

The title compound was prepared according to General Procedure A utilizing 0.402 mmol of 4-methoxypyridin-2-amine. The material was purified by reverse phase column chromatography, eluting with 30-70% methanol in water to give the title compound as a light orange solid (33 mg, 24%). LCMS [M+H]^+^ 335.1 m/z; ^1^H NMR (500 MHz, Chloroform-*d*) δ 9.19 (s, 1H), 8.48 (d, *J* = 4.7 Hz, 1H), 8.26 (d, *J* = 7.9 Hz, 1H), 7.66 (d, *J* = 7.4 Hz, 1H), 7.30 – 7.27 (m, 1H), 7.13 (dd, *J* = 11.5, 8.1 Hz, 1H), 6.91 (d, *J* = 2.4 Hz, 1H), 6.86 (t, *J* = 7.7 Hz, 1H), 6.79 (q, *J* = 7.0 Hz, 1H), 6.52 (dd, *J* = 7.3, 2.4 Hz, 1H), 6.28 (t, *J* = 8.4 Hz, 1H), 5.89 (d, *J* = 3.2 Hz, 1H), 3.88 (s, 3H).

*2-Fluoro-N-[7-methoxy-2-(pyridin-2-yl)imidazo[1,2-a]pyridin-3-yl]benzamide* (**3h**)

The title compound was prepared according to General Procedure B utilizing 0.179 mmol of **S2**. The material was purified by dissolving the precipitate in dichloromethane and washing with a 0.5M aqueous HCl solution to give the title compound as a white solid (59 mg, 74%). LCMS [M+H]^+^ 363.1 m/z; ^1^H NMR (500 MHz, Chloroform-*d*) δ 11.23 (d, *J* = 11.8 Hz, 1H), 8.65 – 8.56 (m, 1H), 8.34 (s, 1H), 8.20 (td, *J* = 7.8, 3.9 Hz, 1H), 7.96 (dd, *J* = 7.8, 2.4 Hz, 1H), 7.83 (t, *J* = 7.9 Hz, 1H), 7.66 – 7.56 (m, 1H), 7.34 (t, *J* = 7.6 Hz, 1H), 7.29 (t, *J* = 11.0 Hz, 1H), 7.22 (t, *J* = 6.0 Hz, 1H), 7.14 (s, 1H), 6.71 (d, *J* = 7.5 Hz, 1H), 3.94 (d, *J* = 2.4 Hz, 3H).

*N-(2-Fluorophenyl)-7-methoxy-2-(pyrimidin-2-yl)imidazo[1,2-a]pyridin-3-amine* (**3i**)

The title compound was prepared according to General Procedure A utilizing 0.402 mmol of 4-methoxypyridin-2-amine. The material was purified by reverse phase column chromatography, eluting with 30-70% methanol in water to give the title compound as a light orange solid (20 mg, 15%). LCMS [M+H]^+^ 336.0 m/z; ^1^H NMR (500 MHz, Chloroform-*d*) δ 8.77 (d, *J* = 4.8 Hz, 2H), 7.73 (s, 1H), 7.55 (d, *J* = 7.5 Hz, 1H), 7.17 – 7.07 (m, 2H), 6.96 (s, 1H), 6.92 – 6.82 (m, 2H), 6.55 (dd, *J* = 7.6, 2.2 Hz, 1H), 6.26 – 6.18 (m, 1H), 3.89 (s, 3H).

*5-Fluoro-N-[7-methoxy-2-(pyridin-2-yl)imidazo[1,2-a]pyridin-3-yl]pyridine-2-carboxamide* (**3k**)

The title compound was prepared according to General Procedure B utilizing 0.175 mmol of **S2**. The title compound was isolated as a light-yellow solid (57 mg, 90%). LCMS [M+H]^+^ 364.0 m/z; ^1^H NMR (500 MHz, Chloroform-*d*) δ 11.94 (s, 1H), 8.67 – 8.59 (m, 2H), 8.32 (dd, *J* = 8.7, 4.5 Hz, 1H), 8.18 (d, *J* = 7.9 Hz, 1H), 8.03 (d, *J* = 7.6 Hz, 1H), 7.78 (td, *J* = 7.8, 1.8 Hz, 1H), 7.61 (td, *J* = 8.3, 2.8 Hz, 1H), 7.16 (ddd, *J* = 7.5, 4.9, 1.2 Hz, 1H), 6.92 (s, 1H), 6.59 (dd, *J* = 7.6, 2.4 Hz, 1H), 3.89 (s, 3H).

*3-Fluoro-N-[7-methoxy-2-(pyridin-2-yl)imidazo[1,2-a]pyridin-3-yl]benzamide* (**3l**)

The title compound was prepared according to General Procedure B utilizing 0.179 mmol of **S2**. The material was purified by dissolving the precipitate in dichloromethane and washing with a 0.5M aqueous HCl solution to give the title compound as a light-yellow solid (32.9 mg, 51%). LCMS [M+H]^+^ 363.0 m/z; ^1^H NMR (500 MHz, Chloroform-*d*) δ 11.70 (s, 1H), 8.42 (d, *J* = 4.7 Hz, 1H), 8.23 (d, *J* = 7.9 Hz, 2H), 8.02 (d, *J* = 7.6 Hz, 1H), 7.98 (d, *J* = 9.4 Hz, 1H), 7.76 (t, *J* = 7.7 Hz, 1H), 7.61 (td, *J* = 7.9, 5.6 Hz, 1H), 7.40 – 7.31 (m, 2H), 7.17 (t, *J* = 6.2 Hz, 1H), 6.78 – 6.70 (m, 1H), 3.90 (s, 3H).

*N-(tert-Butyl)-7-methoxy-2-(pyridin-2-yl)imidazo[1,2-a]pyridin-3-amine* (**S1**)

The title compound was prepared according to General Procedure A utilizing 0.402 mmol of 4-methoxypyridin-2-amine. The material was purified by reverse phase column chromatography, eluting with 30-70% methanol in water to give the title compound as an orange oil (118 mg, 99%). LCMS [M+H]^+^ 297.1 m/z; ^1^H NMR (500 MHz, Chloroform-*d*) δ 8.54 (ddd, *J* = 4.9, 1.9, 1.0 Hz, 1H), 8.12 – 8.08 (m, 2H), 7.73 (td, *J* = 7.8, 1.8 Hz, 1H), 7.12 (ddd, *J* = 7.5, 4.8, 1.2 Hz, 1H), 6.78 (d, *J* = 2.3 Hz, 1H), 6.46 (dd, *J* = 7.6, 2.4 Hz, 1H), 5.36 (s, 1H), 3.85 (s, 3H), 1.13 (s, 9H).

*7-Methoxy-2-(pyridin-2-yl)imidazo[1,2-a]pyridin-3-amine* (**S2**)

Compound **S1** (340 mg, 1.15 mmol) was suspended in 20 mL of hydrobromic acid 3M and stirred at 110 °C for 3h. The reaction was quenched using 11 mL NaOH 5M obtained a yellow precipitate that was filtered as pure compound (220 mg, 80%). LCMS [M+H]^+^ 241.1 m/z; ^1^H NMR (500 MHz, Chloroform-*d*) δ 8.53 (d, *J* = 4.8 Hz, 1H), 8.08 (d, *J* = 8.0 Hz, 1H), 7.77 – 7.68 (m, 1H), 7.63 (d, *J* = 7.5 Hz, 1H), 7.06 (t, *J* = 6.2 Hz, 1H), 6.83 – 6.74 (m, 1H), 6.51 (dd, *J* = 7.6, 2.3 Hz, 1H), 5.22 (s, 2H), 3.85 (s, 3H).

## Scheme 3. Synthesis of 4,5-dimethyl-1*H*-pyrazol-3-amine derivatives.

*^a^Reagents and reaction conditions:* (i) paraformaldehyde, DMSO, rt, 72 h; (ii) sodium sulfide nonahydrate, sulfur, H_2_O, EtOH, Δ, 2.5 h; (iii) a) trifluoroacetic acid, n-butyl nitrite, 1,2-dichloroethane 0-5 °C, 1 h; b) 1,2-dichloroethane, 75 °C, 1 h; (iv) 4,5-dimethyl-1*H*-pyrazol-3-amine, NaOtBu, Pd_2_dba_3_, *t*BuXPhos, tBuOH, 100 °C, 18 h; (v) sodium hydride, iodomethane, THF, rt, 24 h; (vi) 4,5-dimethyl-1*H*-pyrazol-3-amine, NaOtBu, Pd_2_dba_3_, BrettPhos, tBuOH, 100 °C, 18 h.

*2-(2-Chloro-6-nitrophenyl)ethan-1-ol* (**S3**) [2]

The title compound was prepared according to a literature procedure wherein, 2-chloro-6-nitrotoluene (1.0 g, 5.83 mmol) and paraformaldehyde (175 mg) were combined in DMSO (875 µL) and potassium hydroxide (10 mg in 150 µL ethanol) was added. Upon addition, the reaction mixture temporarily went purple and ~30 mins after completion of addition all the solids were dissolved. The reaction was left to stir at ambient temperature for 72 h. The reaction mixture was diluted with water, acidified by addition of 6.0M aqueous hydrochloric acid and extracted with diethyl ether. The organic layers were combined and dried with magnesium sulphate. All the volatiles were removed *in vacuo* and the crude material was purified by column chromatography, eluting with 15% ethyl acetate/hexanes to obtain the title compound as a colourless solid (678 mg, 58%). ^1^H NMR (500 MHz, chloroform-*d*) δ ppm 7.72 (dd, *J*=8.30, 0.98 Hz, 1 H), 7.64 (dd, *J*=8.30, 0.98 Hz, 1 H), 7.34 (t, *J*=8.05 Hz, 1 H), 3.97 (t, *J*=6.83 Hz, 2 H), 3.29 (t, *J*=6.83 Hz, 2 H), 1.73 (br. s., 1 H).

Note: the product does not ionise by LCMS.

*2-(2-Amino-6-chlorophenyl)ethan-1-ol* (**S4**) [2]

The title compound was prepared according to a literature procedure wherein, sodium sulfide nonahydrate (1.62 g, 6.73 mmol) and sulfur (216 mg, 6.73 mmol) were dissolved in 3.5 mL water and 1.7 mL ethanol (total: 5.2 mL, 1.3M). To this solution was added **S3** (678 mg, 3.36 mmol) portion wise. Once the addition was complete, the reaction mixture was heated to reflux for 2.5 h. The progress of the reaction was monitored by LCMS analysis and, once complete, all the volatiles were removed *in vacuo*. The crude residue was then diluted with water and the product was extracted with diethyl ether, the organic phase was collected, washed with water and dried with magnesium sulphate and all of volatiles were removed. The crude material was purified by column chromatography, eluting with 2% methanol/dichloromethane to obtain the title compound as an orange solid (434 mg, 75%). LCMS [M+H]^+^ 171.8 (^35^Cl), 173.8 (^37^Cl) m/z; ^1^H NMR (500 MHz, chloroform-*d*) δ ppm 6.97 (t, *J*=8.30 Hz, 1 H), 6.84 (dd, *J*=7.81, 0.98 Hz, 1 H), 6.62 (d, *J*=7.81 Hz, 1 H), 3.95 (t, *J*=5.86 Hz, 3 H), 3.06 - 3.52 (m, 2 H), 3.01 (t, *J*=6.10 Hz, 2 H); ^1^H NMR (500 MHz, DMSO-*d*_6_) δ ppm 6.87 (t, *J*=8.05 Hz, 1 H), 6.58 (d, *J*=7.81 Hz, 2 H), 5.18 (s, 2 H), 4.75 (t, *J*=5.37 Hz, 1 H), 3.51 (q, *J*=6.80 Hz, 2 H), 2.79 (t, *J*=7.08 Hz, 2 H).

*4-Chloro-2,3-dihydrobenzofuran* (**S5**) [3]

The title compound was prepared according to a literature procedure wherein, a solution of **S4** (434 mg, 2.53 mmol) in 1,2-dichloroethane (1.4 mL, 1.8 M) was added at 0-5 °C to trifluoroacetic acid (387 µL, 5.06 mmol). n-Butyl nitrite (95%, 342 µL, 2.78 mmol) was then added over a period of 10 mins while the temperature was maintained at 0-5 °C and stirred after completion of the addition for 1 h at this temperature. The resulting diazonium salt was not isolated and reacted directly.

The diazonium salt solution was added over a period of 10 mins to 1,2-dichloroethane (2 mL) at ~75 °C and stirred for 1 h at this temperature after completion of addition, the reaction mixture was cooled to ambient temperature before it was washed with a 1M aqueous hydrochloric acid solution. The organic phase was collected, dried over magnesium sulphate, filtered and concentrated *in vacuo*. The crude material was purified by column chromatography, eluting with 10% ethyl acetate/hexanes to obtain the title compound as a dark-yellow oil (224 mg, 57%). ^1^H NMR (500 MHz, chloroform-*d*) δ ppm 7.05 (t, *J*=8.05 Hz, 1 H), 6.84 (d, *J*=7.81 Hz, 1 H), 6.68 (d, *J*=7.81 Hz, 1 H), 4.62 (t, *J*=8.78 Hz, 2 H), 3.26 (t, *J*=8.78 Hz, 2 H).

Note: the product does not ionize by LCMS.

N*-(2,3-Dihydrobenzofuran-4-yl)-3,4-dimethyl-1*H*-pyrazol-5-amine* (**S6**)

Compound **S5** (50 mg, 0.323 mmol), sodium tert-butoxide (37 mg, 0.388 mmol) and 4,5-dimethyl-1*H*-pyrazol-3-amine (43 mg, 0.388 mmol) were combined in tert-butanol (1.30 mL, 0.25M). The reaction mixture was degassed for 2 mins. Pd_2_dba_3_ (6 mg, 0.006 mmol) and *t*BuXPhos (5 mg, 0.013 mmol) were added to the reaction mixture which was again degassed. The reaction mixture was heated to 100 °C on the shaker plate overnight. An LCMS of the crude reaction mixture indicated a mass consistent with the desired product was present. The reaction mixture was filtered through celite and the crude material was purified by column chromatography, eluting with 40-60% ethyl acetate/hexanes. The title compound was obtained as a pale-pink solid (57 mg, 77%). LCMS [M+H]^+^ 230.1 m/z; ^1^H NMR (500 MHz, DMSO-*d*_6_) δ ppm 11.77 (br. s., 1 H), 6.97 (br. s., 1 H), 6.81 (t, *J*=8.05 Hz, 1 H), 6.40 (d, *J*=6.34 Hz, 1 H), 6.11 (d, *J*=7.81 Hz, 1 H), 4.47 (t, *J*=8.78 Hz, 2 H), 3.00 (t, *J*=8.05 Hz, 2 H), 2.12 (s, 3 H), 1.76 (s, 3 H); ^1^H NMR (500 MHz, CHLOROFORM-*d*) δ ppm 7.00 (t, *J*=8.05 Hz, 1 H), 6.61 (d, *J*=8.30 Hz, 1 H), 6.36 (d, *J*=7.81 Hz, 1 H), 5.34 (br. s., 1 H), 4.61 (t, *J*=8.78 Hz, 2 H), 3.07 (t, *J*=8.54 Hz, 2 H), 2.22 (s, 3 H), 1.86 (s, 3 H).

*4-Bromo-2-methylisoindolin-1-one* (**S7**)

4-Bromoisoindolin-1-one (250 mg, 1.18 mmol) was added to a cooled solution of sodium hydride (52 mg, 1.30mmol) in THF (7 mL, 0.16M). After 45 mins iodomethane (294 µL, 4.72 mmol) was added to the reaction mixture which was allowed to gradually warm to ambient temperature. The progress of the reaction was monitored by LCMS and, after 24 h there was only a trace of the starting material evident, and a new peak with a mass consistent with the desired product. The remaining sodium hydride was quenched with the addition of methanol until no more effervescence observed. The crude material was purified by column chromatography, eluting with 25-50% ethyl acetate/hexanes to obtain the title compound as a yellow solid (218 mg, 82%). LCMS [M+H]^+^ 226.0 (^79^Br), 228.0 (^81^Br) m/z; ^1^H NMR (500 MHz, DMSO-*d*_6_) δ ppm 7.80 (d, *J*=7.81 Hz, 1 H), 7.69 (d, *J*=7.32 Hz, 1 H), 7.46 (t, *J*=7.81 Hz, 1 H), 4.41 (s, 2 H), 3.09 (s, 3 H).

*4-((3,4-Dimethyl-1*H*-pyrazol-5-yl)amino)-2-methylisoindolin-1-one* (**S8**)

Compound **S7** (50 mg, 0.222 mmol), cesium carbonate (26 mg, 0.267 mmol) and 4,5-dimethyl-1*H*-pyrazol-3-amine (30 mg, 0.267 mmol) were combined in tert-butanol (888 µl, 0.25M). The reaction mixture was degassed for 2 mins. Pd_2_dba_3_ (4 mg, 0.004 mmol) and BrettPhos (4 mg, 0.009 mmol) were added to the reaction mixture which was again degassed. The reaction mixture was heated to 100 °C overnight. An LCMS of the crude reaction mixture indicated a mass consistent with the desired product was present. The reaction mixture was filtered through celite and the crude material was purified by column chromatography, eluting with 50% ethyl acetate/hexanes and then switching to 100%-95% ethyl acetate/methanol to obtain the title compound. The material was further purified by column chromatography, eluting with 2-5% methanol (+5% ammonium hydroxide)/dichloromethane to obtain the title compound as a pale-yellow solid (10 mg, 18%). LCMS [M+H]^+^ 257.1 m/z; 1H NMR ^1^H NMR (500 MHz, methanol-*d*_4_) δ ppm 7.26 (t, *J*=7.81 Hz, 1 H), 7.16 (d, *J*=7.32 Hz, 1 H), 6.94 (d, *J*=3.90 Hz, 1 H), 4.27 (s, 2 H), 3.17 (s, 3 H), 2.22 (s, 3 H), 1.85 (s, 3 H).

## Scheme 4. Synthesis of 4,5-dimethyl-1*H*-pyrazol-3-amine derivatives.

*^a^Reagents and reaction conditions:* (i) dioxane, aqueous lithium hydroxide, rt, 4 h; (ii) (*S*)-2-amino-2-phenylethan-1-ol, HBTU, diisopropylethylamine, DMF, rt, 1-18 h; (iii) K_2_CO_3_, PdCl_2_(dppf)·CH_2_Cl_2_, 3:1 dioxane:water, microwave, 130 °C, 30 mins; (iv) 2.0 M aqueous lithium hydroxide, 2:1 THF:water, microwave, 150 °C, 2-7 mins.

*5-Bromo-1H-pyrazole-3-carboxylic acid* (**S9**)

Methyl 5-bromo-1*H*-pyrazole-3-carboxylate (220 mg, 1.07 mmol) was suspended in dioxane (4 mL) and an aqueous solution of lithium hydroxide (5.4 mL, 2.0 M, 10.7 mmol) was added and the reaction was stirred at ambient temperature for 4 h. The reaction was monitored by LCMS analysis and, once complete, the reaction mixture was diluted with water and the organic layer evaporated *in vacuo*. The aqueous layer was washed twice with dichloromethane and then acidified to pH 2.0 with concentrated hydrochloric acid. The product was extracted into ethyl acetate, and the combined organic layers washed with brine dried over sodium sulfate, filtered and concentrated *in vacuo* to provide the desired material as a white solid (205 mg, quantitative). LCMS [M+H]^+^ 191.0 m/z (Br^79^), 192.8 m/z (Br^81^); ^1^H NMR (500 MHz, DMSO-*d*_6_) δ ppm 6.87 (s, 1 H). *The -NH and carboxylic acid protons are not observed.

*(S)-5-Bromo-N-(2-hydroxy-1-phenylethyl)-1H-pyrazole-3-carboxamide* (**S10**)

5-Bromo-1*H*-pyrazole-3-carboxylic acid (**S9**; 200 mg, 1.05 mmol) was coupled to (*S*)-2-amino-2-phenylethan-1-ol (155.2 mg, 1.13 mmol) and HBTU (428.9 mg, 1.13 mmol) were stirred in DMF (10 ml). Diisopropylethylamine (492.5 µL, 2.83 mmol) was added and mixture was stirred at ambient temperature. After 1 h the reaction had completed and the reaction mixture was diluted with water, and the aqueous solution was extracted with ethyl acetate (3 × 20 mL), and the combined organic layers were washed with brine, dried over sodium sulfate and concentrated *in vacuo*. The crude material was purified by flash chromatography over silica, eluting with 60-80% ethyl acetate/hexanes to afford the title compound as a white powder (291 mg, 99%). [α]_D_^25^ –43 (*c* 0.53, CH_3_OH). LCMS [M+H]^+^ 310.0 m/z (Br^79^), 312.0 m/z (Br^81^); ^1^H NMR (500 MHz, METHANOL-*d_4_*) δ ppm 7.37 - 7.40 (m, 2 H), 7.31 - 7.36 (m, 2 H), 7.23 - 7.29 (m, 1 H), 6.92 (br. s., 1 H), 5.15 (t, *J*=6.3 Hz, 1 H), 3.79 - 3.88 (m, 2 H).

*(S)-N-(2-Hydroxy-1-phenylethyl)-5-(1-tosyl-1H-pyrrolo[2,3-b]pyridin-3-yl)-1H-pyrazole-3-carboxamide* (**S11**)

1-Tosyl-1*H*-pyrrolo[2,3-*b*]pyridine-3-boronic acid, pinacol ester (80 mg, 0.200 mmol), **S10** (74.8 mg, 0.241 mmol), [1,1′-bis(diphenylphosphino)ferrocene]dichloropalladium(II) (11.6 mg, 10 µmol), potassium carbonate (62.5 mg, 0.452 mmol) were added to a microwave vial and the solids purged with nitrogen. To this, a solution of 3:1 dioxane:water (2 mL, 0.1 M) was added and the solution degassed for 10 mins. The reaction was undertaken in the microwave for 30 mins (130 °C, high absorbance). After 30 mins, it was evident by LCMS the reaction was complete and the reaction mixture was diluted with methanol, filtered through celite, and the filtrate concentrated *in vacuo*. The crude material was purified by flash chromatography over silica, eluting with 2-3% methanol/dichloromethane to afford the title compound as a pale-pink semi-solid (23 mg, 23%). [α]_D_^18^ –26 (*c* 1.56, CH_3_OH). LCMS [M+H]^+^ 502.0 m/z; ^1^H NMR (500 MHz, methanol-*d_4_*) δ ppm 8.38 - 8.46 (m, 1 H), 8.22 - 8.30 (m, 1 H), 8.05 (d, *J*=8.3 Hz, 2 H), 7.41 - 7.46 (m, 2 H), 7.32 - 7.40 (m, 7 H), 7.25 - 7.29 (m, 1 H), 5.20 (t, *J*=5.9 Hz, 1 H), 3.87 - 3.91 (m, 2 H), 2.37 (s, 3 H).

*(S)-N-(2-Hydroxy-1-phenylethyl)-5-(1H-pyrrolo[2,3-b]pyridin-3-yl)-1H-pyrazole-3-carboxamide* (**S12**)

Compound **S11** was dissolved in a 2:1 tetrahydrofuran:water (0.025 M) mixture in a microwave vial. To this an aqueous solution of lithium hydroxide (2.0 M, 10 eq.) was added, and the reaction subjected to microwave irradiation for 7 minutes at 150 °C (high absorbance). The reaction was monitored by LCMS and, once complete, the reaction mixture was diluted with ethyl acetate, filtered, washed with an aqueous hydrochloric acid solution (1M), brine, dried over sodium sulfate, filtered and organics concentrated *in vacuo*. The crude material was purified by column chromatography, eluting with 7% methanol/dichloromethane to afford the title compound as a white solid (8 mg, 51%). LCMS [M+H]^+^ 348.1 m/z; ^1^H NMR (500 MHz, methanol-*d_4_*) δ ppm 8.26 - 8.35 (m, 2 H), 7.81 (s, 1 H), 7.42 - 7.46 (m, 2 H), 7.33 - 7.39 (m, 2 H), 7.23 - 7.30 (m, 2 H), 7.05 (br. s., 1 H), 5.20 (t, *J*=5.9 Hz, 1 H), 3.86 - 3.92 (m, 2 H).

(*S*)-6-Bromo-*N*-(2-hydroxy-1-phenylethyl)nicotinamide (**S13**)

6-Bromonicotinic acid (150 mg, 0.743 mmol), (S)-2-amino-2-phenylethan-1-ol (122.2 mg, 0.297 mmol) and HBTU (337.9 mg, 0.891 mmol) were stirred in DMF (7.5 ml). Diisopropylethylamine (388 µL, 2.23 mmol) was added 15 mins later and the mixture was stirred at ambient temperature. After 18 h the reaction had completed and the reaction mixture was diluted with water, and the aqueous solution was extracted with ethyl acetate (3 × 20 mL), and the combined organic layers were washed with brine, dried over sodium sulfate and concentrated *in vacuo*. The crude material was purified by flash chromatography over silica, eluting with 50% ethyl acetate/hexanes to afford the title compound as a white solid (220 mg, 69%). [α]_D_^25^ –38 (*c* 0.52, CH_3_OH). LCMS [M+H]^+^ 321.1 m/z (Br^79^), 323.1 m/z (Br^81^); ^1^H NMR (500 MHz, DMSO-*d*_6_) δ ppm 9.00 (d, *J*=8.3 Hz, 1 H), 8.86 (d, *J*=2.4 Hz, 1 H), 8.18 (dd, *J*=8.3, 2.4 Hz, 1 H), 7.80 (d, *J*=8.3 Hz, 1 H), 7.39 (d, *J*=7.3 Hz, 2 H), 7.32 (t, *J*=7.3 Hz, 2 H), 7.24 (t, *J*=7.3 Hz, 1 H), 5.04 - 5.10 (m, 1 H), 4.98 (t, *J*=5.9 Hz, 1 H), 3.60 - 3.74 (m, 2 H).

(*S*)-*N*-(2-Hydroxy-1-phenylethyl)-6-(1-tosyl-1*H*-pyrrolo[2,3-*b*]pyridin-3-yl)nicotinamide (**S14**)

1-Tosyl-1*H*-pyrrolo[2,3-*b*]pyridine-3-boronic acid, pinacol ester (100 mg, 0.251 mmol), **S13** (80.6 mg, 0.251 mmol), tetrakis(triphenylphosphine)palladium(0) (14.5 mg, 0.013 mmol), potassium carbonate (78.1 mg, 0.565 mmol) were added to a microwave vial and the solids purged with nitrogen. To this a solution of 3:1 dioxane:water (2.5 mL, 0.1 M) was added and the solution degassed for 10 mins. The reaction was undertaken in the microwave for 30 mins (130 °C, high). After 10 mins, it was evident by LCMS the reaction was complete and the reaction mixture was diluted with methanol, filtered through celite, and the filtrate concentrated *in vacuo*. The crude material was purified by flash chromatography over silica, eluting with 0-5% methanol/dichloromethane to afford the title compound as a deep pink foam (77 mg, 60%). LCMS [M+H]^+^ 513.1 m/z; ^1^H NMR (500 MHz, DMSO-*d*_6_) δ ppm 9.10 (d, *J*=2.0 Hz, 1 H), 8.90 (d, *J*=8.3 Hz, 1 H), 8.85 (dd, *J*=7.8, 1.2 Hz, 1 H), 8.76 (s, 1 H), 8.40 (d, *J*=4.6 Hz, 1 H), 8.30 (dd, *J*=8.3, 2.0 Hz, 1 H), 8.22 (d, *J*=8.3 Hz, 1 H), 8.03 (d, *J*=8.3 Hz, 2 H), 7.34 - 7.44 (m, 5 H), 7.26 - 7.33 (m, 2 H), 7.17 - 7.24 (m, 1 H), 5.03 - 5.11 (m, 1 H), 4.95 (t, *J*=5.9 Hz, 1 H), 3.59 - 3.74 (m, 2 H), 2.30 (s, 3 H).

*(S)-N-(2-Hydroxy-1-phenylethyl)-6-(1H-pyrrolo[2,3-b]pyridin-3-yl)nicotinamide* (**S15**)

Compound **S14** (77 mg, 0.150 mmol) was dissolved in a 2:1 tetrahydrofuran:water (0.025 M) mixture in a microwave vial. To this an aqueous solution of lithium hydroxide (2.0 M, 10 eq.) was added, and the reaction subjected to microwave irradiation for 2 minutes at 150 °C (high absorbance). The reaction was monitored by LCMS and, once complete, the reaction mixture was diluted with ethyl acetate, filtered, washed with an aqueous hydrochloric acid solution (1M), brine, dried over sodium sulfate, filtered and organics concentrated *in vacuo*. The crude material was purified by column chromatography, eluting with 5% methanol/dichloromethane to afford the title compound as a pale-yellow solid (21 mg, 39%). [α]_D_^23^ –86 (*c* 0.53, CH_3_OH). LCMS [M+H]^+^ 359.1 m/z; ^1^H NMR (500 MHz, DMSO-*d*_6_) δ ppm 12.20 (br. s., 1 H), 9.10 (d, J=2.4 Hz, 1 H), 8.80 - 8.85 (m, 2 H), 8.39 (d, *J*=2.4 Hz, 1 H), 8.30 (dd, *J*=4.6, 1.2 Hz, 1 H), 8.23 (dd, *J*=8.3, 2.4 Hz, 1 H), 8.00 (d, *J*=8.3 Hz, 1 H), 7.40 - 7.44 (m, 2 H), 7.31 - 7.36 (m, 2 H), 7.20 - 7.27 (m, 2 H), 5.08 - 5.13 (m, 1 H), 4.98 (t, *J*=5.9 Hz, 1 H), 3.71 - 3.77 (m, 1 H), 3.63 - 3.70 (m, 1 H).

## Scheme 5. Synthesis of 4,5-dimethyl-1*H*-pyrazol-3-amine derivatives.

*^a^Reagents and reaction conditions:* (i) pinacol ester, K_2_CO_3_, PdCl_2_(dppf)·CH_2_Cl_2_, 3:1 dioxane:water, microwave, 150 °C, 30 mins; (ii) Palladium 10 wt% on Carbon, ammonium formate, EtOH, H_2_ (g), 85 °C 2 h; (iii) 4M HCl in dioxane, rt, 3 h.

*Tert-butyl* *5-(4-(pyrazin-2-ylamino)quinolin-7-yl)-3,6-dihydropyridine-1(2H)-carboxylate* (**S16**)

7-Bromo-*N*-(pyrazin-2-yl)quinolin-4-amine (350 mg, 1.16 mmol), 1-boc-5,6-dihydro-2*H*-pyridine-3-boronic acid, pinacol ester (424 mg, 1.37 mmol), K_2_CO_3_ (484 mg, 3.50 mmol), and PdCl_2_(dppf)·CH_2_Cl_2_ (47 mg, 5 mol %) were combined in a microwave vial that was filled with nitrogen and evacuated three times. 3:1 Dioxane:water (0.08 M) was added and the reaction mixture was degassed, then run in the microwave (150 °C, H abs) for 30 minutes. The reaction mixture was diluted with EtOAc, filtered through celite, and concentrated under reduced pressure. The crude material was purified by flash chromatography (100% EtOAc) to afford the title compound as a light-yellow solid (315 mg, 67%). LCMS [M+H]^+^ 404.23 m/z; ^1^H NMR (500 MHz, DMSO-*d*_6_) δ ppm 9.78 (s, 1 H) 8.68 - 8.73 (m, 2 H) 8.47 (d, *J*=8.8 Hz, 1 H) 8.29 - 8.33 (m, 2 H) 8.17 (d, *J*=2.9 Hz, 1 H) 7.85 (br. s, 1 H) 7.81 (d, *J*=10.7 Hz, 1 H) 6.64 (br. s., 1 H) 4.38 (br. s., 2 H) 3.53 (br. s., 2 H) 2.35 (br. s., 2 H) 1.45 (s, 9 H).

*Tert-butyl 3-(4-(pyrazin-2-ylamino)quinolin-7-yl)piperidine-1-carboxylate* (**S17**)

Compound **S16** (315 mg, 0.781 mmol) was dissolved in EtOH (0.05 M) and 10% wt Pd/C (85 mg, 10 mol %) was added. Ammonium formate (320 mg, 5.07 mmol) was added and the reaction was refluxed at 85 °C for 2 h. After cooling to room temperature, the reaction mixture was diluted with EtOAc, filtered through celite, and concentrated under reduced pressure. The crude material was purified by column chromatography 10% methanol/dichloromethane, then repurified by preparative HPLC (99-50% ACN:water) to afford the title compound as a yellow solid (54 mg, 17%). LCMS [M+H]^+^ 406.25 m/z; ^1^H NMR (500 MHz, methanol-*d*_4_) δ ppm 8.66 (s, 2 H) 8.53 (d, *J*=5.9 Hz, 1 H) 8.46 (d, *J*=8.8 Hz, 1 H) 8.39 (d, *J*=1.0 Hz, 1 H) 8.22 (d, *J*=2.0 Hz, 1 H) 7.85 (s, 1 H) 7.67 (d, *J*=5.9 Hz, 1 H) 4.20 - 4.33 (m, 1 H) 4.16 (d, *J*=15.1 Hz, 1 H) 2.93 (br. s., 3 H) 2.15 (d, *J*=11.7 Hz, 1 H) 1.87 (m, *J*=11.2 Hz, 2 H) 1.66 (q, *J*=13.7 Hz, 1 H) 1.48 (s, 9 H).

*7-(Piperidin-3-yl)-N-(pyrazin-2-yl)quinolin-4-amine* (**S18**)

Compound **S17** (99 mg, 0.244 mmol) was taken up in 4M HCl in dioxane (600 µL, 2.4 mmol). The reaction was stirred at room temperature for three hours. All volatiles were removed *in vacuo.* The resulting solid was dissolved in methanol and Si-carbonate was added. After stirring overnight at room temperature, Si-carbonate was removed by filtration and volatiles were again removed *in vacuo*. The crude material was purified by flash chromatography (10-20% 10%NH_4_OH/MeOH:DCM) to afford the title compound as a light yellow solid (52 mg, 69%). LCMS [M+H]^+^ 306.18 m/z; ^1^H NMR (500 MHz, methanol-*d*_4_) δ ppm 8.69 (d, *J*=5.9 Hz, 1 H) 8.66 (s, 1 H) 8.55 (d, *J*=5.4 Hz, 1 H) 8.52 (d, *J*=8.8 Hz, 1 H) 8.39 (dd, *J*=2.4, 1.5 Hz, 1 H) 8.22 (d, *J*=2.9 Hz, 1 H) 7.89 (d, *J*=1.5 Hz, 1 H) 7.69 (dd, *J*=8.8, 1.5 Hz, 1 H) 3.59 (d, *J*=7.8 Hz, 1 H) 3.50 (d, *J*=11.2 Hz, 1 H) 3.25 - 3.30 (m, 2 H) 3.12 (td, *J*=12.7, 2.9 Hz, 1 H) 2.20 (d, *J*=8.8 Hz, 1 H) 2.15 (d, *J*=7.3 Hz, 1 H) 1.98 (t, *J*=10.7 Hz, 2 H).

## Scheme 6. Synthesis of the aminopyrimidine derivative.

*^a^Reagents and reaction conditions:* (i) H_2_O, cat. 12M aqueous HCl, 50 °C for 8 h; (ii) *n*-BuOH, microwave, 150 °C, 1 h.

*7-((2-Chloropyrimidin-4-yl)amino)isoindolin-1-one* (**S19**)

7-Aminoisoindolin-1-one (37 mg, 2450 μmol) and 2,4-dichloropyrimidine (36.8mg, 247umol) were dissolved in 1 mL of H_2_O, and a drop of 12M HCl was added, the reaction was stirred at 50 °C.  After 2 h additional 2,4-dichloropyrimidine (36.8mg, 247umol) was added and the reaction was stirred for an additional 2 h, this process was repeated two additional times for a total reaction time of 8 h. The reaction mixture was then filtered and washed with water, to give the title compound as a buff solid (64 mg, 99%). LCMS [M+H]^+^ 261.0 (^35^Cl), 263.0 (^37^Cl) m/z; 1H NMR (500 MHz, DMSO-d6) δ ppm 4.36 - 4.43 (m, 2 H) 6.98 - 7.08 (m, 1 H) 7.21 - 7.30 (m, 1 H) 7.54 - 7.68 (m, 1 H) 8.21 - 8.36 (m, 2 H) 8.80 - 8.90 (m, 1 H) 10.30 - 10.44 (m, 1 H).

*7-((2-((3-oxo-1,3-dihydroisobenzofuran-5-yl)amino)pyrimidin-4-yl)amino)isoindolin-1-one* (**S20**)

Compound **S19** (64.6 mg, 247.8 μmol) was dissolved with 6-aminoisoindolin-1-one (44.4 mg, 297.4 μmol) in *n*-BuOH and subject to MW irradiation at 150 °C for 1 h. The reaction was cooled to RT and filtered. The precipitate was washed with MeOH, then place on a high vac, to afford the title compound as an off-white solid (35.7 mg, 39 %). LCMS [M+H]^+^ 374.1 m/z; 1H NMR (500 MHz, DMSO-d6) δ ppm 4.29 - 4.46 (m, 2 H) 5.32 - 5.51 (m, 2 H) 6.50 - 6.69 (m, 1 H) 7.20 - 7.35 (m, 1 H) 7.42 - 7.57 (m, 1 H) 7.59 - 7.71 (m, 1 H) 7.85 (br d, J=7.81 Hz, 1 H) 7.97 - 8.33 (m, 3 H) 8.82 (s, 1 H) 10.34 - 10.78 (m, 2 H).

# **Table S1.** Additional compounds not presented in the manuscript.

| **ID** |  | **Project** | **SMILES** | ***A. castellanii* EC_50_ (µM) ± SD.** | ***N. fowleri* EC_50_ (µM)**  **± SD.** | ***B. mandrillaris* EC_50_ (µM) ± SD.** |
| --- | --- | --- | --- | --- | --- | --- |
| NEU-6043 | S6 | 42 | CC1=NNC(NC2=CC=CC3=C2CCO3)=C1C | > 20 | > 20 | > 20 |
| NEU-5839 | S8 | 42 | CN1CC2=C(C=CC=C2NC2=C(C)C(C)=NN2)C1=O | > 20 | > 20 | > 20 |
| NEU-5841 | S12 | 20 | OC[C@@H](NC(=O)C1=NNC(=C1)C1=CNC2=NC=CC=C12)C1=CC=CC=C1 | > 20 | > 20 | > 20 |
| NEU-5504 | S15 | 20 | OC[C@@H](NC(=O)C1=CC=C(N=C1)C1=CNC2=NC=CC=C12)C1=CC=CC=C1 | > 20 | > 20 | > 20 |
| NEU-5980 | S18 | Lapatinib | C1CNCC(C1)C1=CC=C2C(NC3=NC=CN=C3)=CC=NC2=C1 | > 20 | > 20 | > 20 |
| NEU-4802 | S20 | 17 | O=C1NCC2=CC=CC(NC3=NC(NC4=CC5=C(COC5=O)C=C4)=NC=C3)=C12 | > 20 | 3.3 ± 1.0 | > 20 |
| NEU-4854 [4] | S21 | 1 | CN1C=C(NC2=NC(NCC(F)(F)F)=C3N=CNC3=N2)C=N1 | > 20 | > 20 | > 20 |
| NEU-5407 [4] | S22 | 1 | CN1C=C(NC2=NC(N3CCOCC3)=C3N=CNC3=N2)C=N1 | > 20 | > 20 | > 20 |
| NEU-4852 [4] | S23 | 1 | FC1=CC=C(NC2=NC(NCC(F)(F)F)=C3N=CNC3=N2)C=C1 | > 20 | > 20 | > 20 |
| NEU-5308 [4] | S24 | 1 | CN1C=C(NC2=NC(NCCCC(F)(F)F)=C3N=CNC3=N2)C=N1 | > 20 | > 20 | > 20 |
| NEU-4907 [4] | S25 | 1 | C1CN(CCO1)C1=C2N=CNC2=NC(NC2=CSC=C2)=N1 | > 20 | > 20 | > 20 |
| NEU-2611 [5] | S26 | 17 | CC(C)(C)C1=CC=C(NC2=NC(=CC=N2)C2=C3C=CC=NN3N=C2C2=CC=C(C=C2)C(F)(F)F)C=C1 | > 20 | > 20 | > 20 |
| NEU-2614 [5] | S27 | 17 | CC(C)(O)C1=NN2N=CC=CC2=C1C1=CC=NC(NC2=CC=C(C=C2)C(N)=O)=N1 | > 20 | > 20 | > 20 |
| NEU-4902 [5] | S28 | 17 | C1=NN2N=CC=CC2=C1C1=CC=NC=C1 | > 20 | > 20 | > 20 |
| NEU-4971 [5] | S29 | 17 | OCC1=NN2N=C(C=CC2=C1C1=NC(NC2=CC(=CC=C2)C#N)=NC=C1)N1CCOCC1 | > 20 | > 20 | > 20 |
| NEU-2118 [6] | S30 | 24 | CN(C)S(=O)(=O)C1=CC=C(N\N=C2/C(=O)NC3=CC=CC=C23)C=C1 | > 20 | > 20 | > 20 |
| NEU-5903 [7] | S31 | 34 | C1CC(CCO1)N1C=C(C=N1)C1=CN=C2NC=C(C2=C1)C1=CC=NC=C1 | > 20 | > 20 | > 20 |
| NEU-4438 [8] | S32 | Lapatinib | CN1C=C\C(=N/C2=NC=CN=C2)C2=CC=C(C=C12)C1=CN=C(N=C1)N1CCCN(C)CC | > 20 | > 20 | > 20 |
| NEU-4363 [8] | S33 | Lapatinib | CN1CCCN(CC1)C1=NC=C(C=N1)C1=CC=C2C(NC3=NC=CN=C3)=CC=NC2=C1 | > 20 | > 20 | > 20 |
| NEU-4443 [8] | S34 | Lapatinib | CN1C[C@@H]2C[C@H]1CN2C1=NC=C(C=N1)C1=CC=C2C(NC3=NC=CN=C3)=CC=NC2=C1 | > 20 | > 20 | > 20 |
| NEU-6000 [8] | S35 | Lapatinib | CN1CCC2(CC1)CCN(CC2)C1=NC=C(C=N1)C1=CC=C2C(NC3=NC=CN=C3)=CC=NC2=C1 | > 20 | > 20 | > 20 |
| NEU-4955 [8] | S36 | Lapatinib | CN1C[C@H]2C[C@@H]1CN2C1=NC=C(C=N1)C1=CC=C2C(NC3=NC=CN=C3)=CC=NC2=C1 | > 20 | > 20 | > 20 |
| NEU-5136 [9] | S37 | Curaxin | CN(C)C1=CC=C(N2C3=CC=C(C(C)=O)C=C3C3=C2C=CC(C(C)=O)=C3)C=C1 | > 20 | > 20 | > 20 |
| NEU-5765 [9] | S38 | Curaxin | CC(=O)C1=CC2=C(C=C1)N(CCN1CCNCC1)C1=CC=C(C(C)=O)C=C21 | > 20 | > 20 | > 20 |
| NEU-6023 [9] | S39 | Curaxin | CC(=O)C1=CC2=C(C=C1)N(CCN1CC(F)C1)C1=CC=C(C(C)=O)C=C21 | > 20 | > 20 | > 20 |
| NEU-6071 [9] | S40 | Curaxin | CC(C)OCCN1C2=C(C=C(C(C)=O)C=C2)C2=C1C=CC(C(C)=O)=C2 | > 20 | > 20 | > 20 |
| NEU-6040 [9] | S41 | Curaxin | CN(C)C1CN(CCN2C3=CC=C(C(C)=O)C=C3C3=C2C=CC(C(C)=O)=C3)C1 | > 20 | > 20 | > 20 |
| NEU-6036 [9] | S42 | Curaxin | CN(C)CCNCCN1C2=C(C=C(C(C)=O)C=C2)C2=C1C=CC(C(C)=O)=C2 | > 20 | > 20 | > 20 |
| NEU-5882 [9] | S43 | Curaxin | CC(=O)C1=CC2=C(C=C1)N(CCN1C=CN=C1)C1=CC=C(C(C)=O)C=C21 | > 20 | > 20 | > 20 |
| Azithromycin | |  |  | 0.26 ± 0.14 | 0.02 ± 0.01 | > 20 |
| Chlorhexidine | |  |  | 11 ± 3.5 | 5.8 ± 0.22 | 1.6 ± 0.17 |
| Simvastatin | |  |  | *nt* | *nt* | 6.9 ± 0.04 |

## Table S2. Additional cytotoxicity data for all tested compounds.

| **ID** | **NEU Number** | **MRC5 TC_50_ ± Standard Deviation** | **L6 TC_50_ ± Standard Deviation** | **THP-1 TC_50_ ± Standard Deviation** | **HepG2 TC_50_ (R^2^)** |
| --- | --- | --- | --- | --- | --- |
| **1a** | NEU-2101 | > 50 | 32 ± 2.5 | > 50 |  |
| **1b** | NEU-2102 | > 50 | 1.0 ± 0.14 | 4.6 ± 0.60 |  |
| **1c** | NEU-5126 | > 50 | 6.3 ± 0.55 | 5.0 ± 0.11 |  |
| **1d** | NEU-6444 | > 50 | > 17 ± 0.052 | > 50 |  |
| **1e** | NEU-4461 | > 50 | > 50 | > 50 |  |
| **2a** | NEU-4408 | > 50 | 3.2 ± 0.077 | > 26 |  |
| **2b** | NEU-4932 | > 50 | < 0.62 | < 0.62 |  |
| **2c** | NEU-5135 | > 50 | 22 ± 2.1 | > 50 |  |
| **2d** | NEU-5298 | > 50 | < 0.62 | < 0.62 |  |
| **2e** | NEU-5299 | > 50 | > 50 | > 50 |  |
| **2f** | NEU-5053 | > 50 | > 50 | > 50 |  |
| **2g** | NEU-5297 | 19 ± 1.0 | 28 ± 1.1 | > 50 |  |
| **2h** | NEU-4393 | > 50 | 4.2 ± 0.89 | 5.0 ± 0.84 |  |
| **2i** | NEU-4407 | > 50 | 48 ± 9.4 | > 50 |  |
| **2j** | NEU-4426 | > 39 ± 18 | 4.1 ± 0.94 | 5.2 ± 0.037 |  |
| **2k** | NEU-4803 | > 50 | 9.9 ± 1.7 | 7.9 ± 0.63 |  |
| **2l** | NEU-4925 | > 50 | 14 ± 1.1 | > 50 |  |
| **2m** | NEU-4926 | > 50 |  | > 50 |  |
| **2n** | NEU-4934 | 33 ± 2.0 | 19 ± 1.1 | 48 ± 4.8 |  |
| **2o** | NEU-4936 | > 50 | 14 ± 0.75 | > 50 |  |
| **2p** | NEU-4937 | > 50 | 5.5 ± 1.1 | 0.87 ± 0.015 |  |
| **2q** | NEU-5010 | > 50 | 50 ± 0 | > 50 |  |
| **2r** | NEU-5130 | > 50 | 31 ± 2.1 | > 50 |  |
| **2s** | NEU-5395 | > 50 | 31 ± 1.3 | 12 ± 2.8 |  |
| **2t** | NEU-5042 | > 50 | 3.7 ± 0.46 | 2.4 ± 0.18 |  |
| **2u** | NEU-5396 | > 50 | > 50 | > 50 |  |
| **3a** | NEU-6085 | 64^a^ |  |  |  |
| **3b** | NEU-6109 | 64^a^ |  |  |  |
| **3c** | NEU-6108 | 64^a^ |  |  |  |
| **3d** | NEU-6084 | 64^a^ |  |  |  |
| **3e** | NEU-5570 |  |  |  |  |
| **3f** | NEU-6089 | 53^a^ |  |  |  |
| **3g** | NEU-6106 | 64^a^ |  |  |  |
| **3h** | NEU-6104 | 64^a^ |  |  |  |
| **3i** | NEU-6105 | 49^a^ |  |  |  |
| **3j** | NEU-6107 | 46^a^ |  |  |  |
| **3k** | NEU-6101 | 64^a^ |  |  |  |
| **3l** | NEU-6102 | 59^a^ |  |  |  |
| **4a** | NEU-6429 | > 50 | 2.8 ± 0.92 | > 50 |  |
| **4b** | NEU-6445 | > 50 | 11 ± 1.5 | > 50 |  |
| **4c** | NEU-6095 | 13 ± 2.4 | 9.0 ± 0.78 | 30 ± 11 |  |
| **4d** | NEU-4828 | > 50 | 17 ± 2.0 | 38 ± 8.7 |  |
| **4e** | NEU-5054 | > 50 | > 50 | > 50 | > 50 |
| **4f** | NEU-5006 | > 50 | > 50 | > 50 |  |
| **5a** | NEU-5123 | 17 ± 2.4 | < 0.62 | 0.14 ± 0.010 | 19 (0.92) |
| **5b** | NEU-6049 | 5.7 ± 0.45 | < 0.62 | < 0.62 |  |
| **5c** | NEU-1200 | 14 ± 2.4 | < 0.62 | < 0.62 | 13 (0.98) |
| **5d** | NEU-6018 | 1.3 ± 0.048 | < 0.62 | < 0.62 | > 29 |
| **5e** | NEU-5306 | > 5.5 | < 0.62 | < 0.62 |  |
| **6a** | NEU-1106 | 50 | 13 ± 5.1 | > 50 |  |
| **6b** | NEU-4945 | > 50 | > 50 | > 50 |  |
| **6c** | NEU-5516 | > 50 | > 50 | > 50 |  |
| **6d** | NEU-5134 | > 50 | > 50 | > 50 |  |
| **6e** | NEU-4943 | > 50 | > 50 | > 50 |  |
| **7a** | NEU-1060 |  |  |  | 3 (0.91) |
| **7b** | NEU-5972 |  |  |  | > 24 |
| **8a** | NEU-5768 |  |  |  | 0.67 (0.33) |
| **8b** | NEU-5159 |  |  |  |  |
| **8c** | NEU-5161 |  |  |  | 1 (0.57) |
| **8d** | NEU-6060 |  |  |  | 3.1 (0.6) |
| **8e** | NEU-5160 |  |  |  |  |
| **8f** | NEU-5137 |  |  |  | 5.8 (0.92) |
| **8g** | NEU-5879 |  |  |  |  |
| **8h** | NEU-5771 |  |  |  | 0.31 (0.64) |
| **8i** | NEU-6038 |  |  |  | 2.8 (0.93) |
| **8j** | NEU-6058 |  |  |  | 5.9 (0.96) |
| **8k** | NEU-5769 |  |  |  |  |
| **8l** | NEU-6070 |  |  |  | 11 (0.98) |
| **S6** | NEU-6043 | > 50 | > 50 | > 50 |  |
| **S8** | NEU-5839 | > 50 | > 50 | > 50 |  |
| **S12** | NEU-5841 | 42 ± 7.7 | > 17 | 42 ± 11 |  |
| **S15** | NEU-5504 | > 50 | > 50 | 26 ± 2.0 |  |
| **S18** | NEU-5980 |  |  |  | > 6.6 |
| **S20** | NEU-4802 | > 50 | > 50 | > 50 |  |
| **S21** | NEU-4854 | 50 | 28 ± 1.4 | > 50 |  |
| **S22** | NEU-5407 | 19 ± 0.42 | 11 ± 1.0 | 19 ± 0.52 |  |
| **S23** | NEU-4852 | > 50 | > 50 | > 50 |  |
| **S24** | NEU-5308 | 50 ± 0 | > 50 | > 50 |  |
| **S25** | NEU-4907 | > 50 | 12 ± 0.83 | > 50 |  |
| **S26** | NEU-2611 | > 50 | > 50 | > 50 |  |
| **S27** | NEU-2614 | > 50 |  | > 50 |  |
| **S28** | NEU-4902 | > 50 | 45 ± 1.5 | 33 ± 14 |  |
| **S29** | NEU-4971 | > 50 |  | > 50 |  |
| **S30** | NEU-2118 | > 50 |  | > 50 |  |
| **S31** | NEU-5903 | 7.9 ± 2.3 | 5.7 ± 0.49 | 5.8 ± 0.43 |  |
| **S32** | NEU-4438 |  |  |  | 36 |
| **S33** | NEU-4363 |  |  |  | 33 (0.87) |
| **S34** | NEU-4443 |  |  |  | 33 |
| **S35** | NEU-6000 |  |  |  | > 21 |
| **S36** | NEU-4955 |  |  |  | > 37 |
| **S37** | NEU-5136 |  |  |  | 21 (0.94) |
| **S38** | NEU-5765 |  |  |  | 7.1 (0.4) |
| **S39** | NEU-6023 |  |  |  | 17 (0.9) |
| **S40** | NEU-6071 |  |  |  | > 30 |
| **S41** | NEU-6040 |  |  |  | 27 (0.97) |
| **S42** | NEU-6036 |  |  |  | 5.5 (0.98) |
| **S43** | NEU-5882 |  |  |  | > 29 |

# **Figure S1.** Lipophilic ligand efficiency of all active compounds against *Acanthamoeba castellanii*, *Naegleria fowleri*, and *Balamuthia mandrillaris.* LLE calculated as pEC_50_-LogD_7.4_.


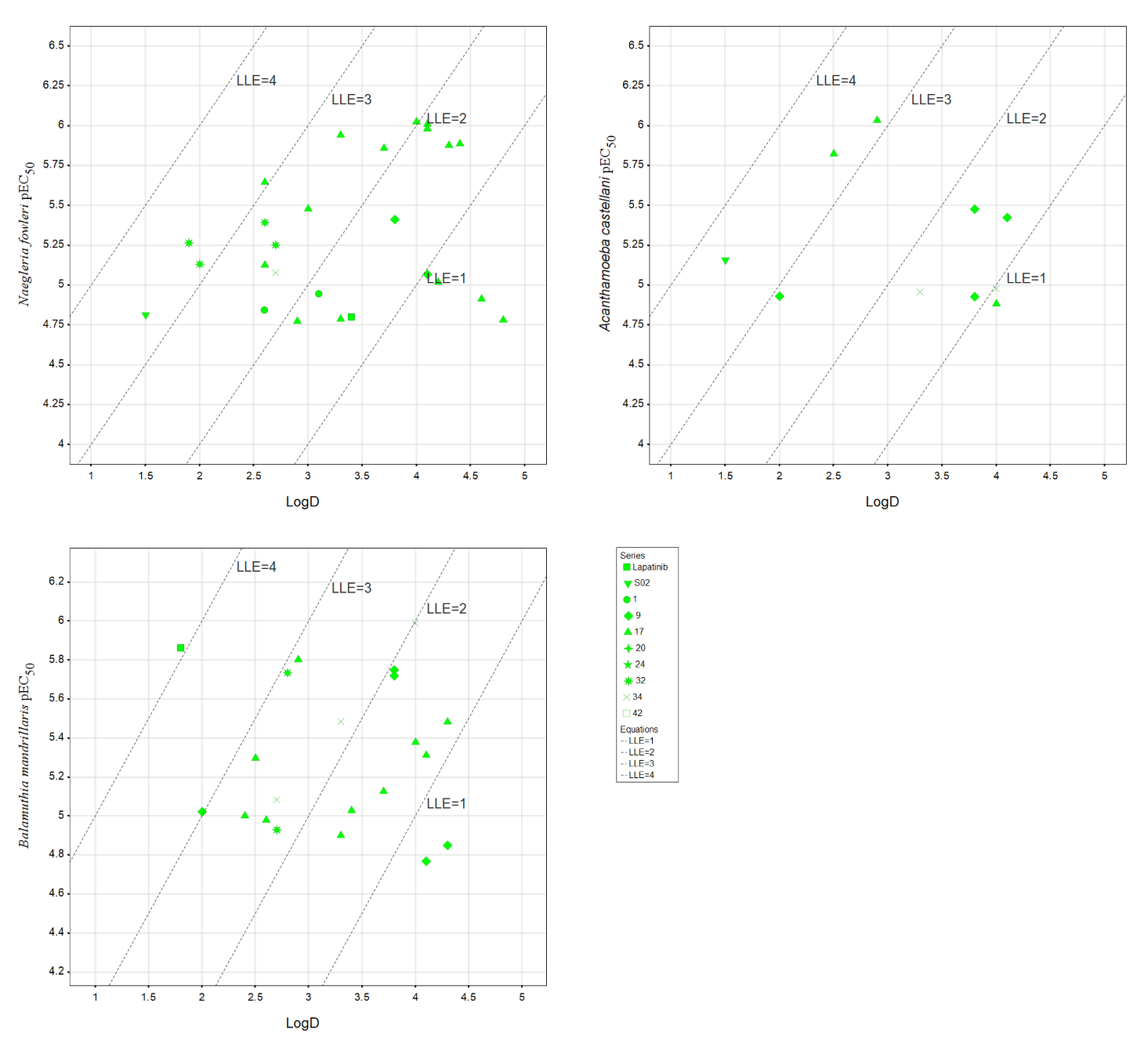


# **References**

1. Gomez-Perez, V., et al., *4-Amino bis-pyridinium derivatives as novel antileishmanial agents.* Antimicrob. Agents Chemother., 2014. **58**(7): p. 4103-4112.

2. Florvall, L., et al., *Selective monoamine oxidase inhibitors. 3. Cyclic compounds related to 4-aminophenethylamine. Preparation and neuron-selective action of some 5-(2-aminoethyl)-2,3-dihydroindoles.* Journal of Medicinal Chemistry, 1986. **29**(8): p. 1406-1412.

3. Gallenkamp, D. and M.J. Ford, *Method for producing 4-substituted 2,3-dihydro-1-benzofuran derivatives by cylisation of 2-(2-diazonium-6-substituted phenyl)ethanol salts*, WIPO, Editor. 2017.

4. Singh, B., et al., *Medicinal Chemistry Optimization of a Diaminopurine Chemotype: Toward a Lead for Trypanosoma brucei Inhibitors.* Journal of Medicinal Chemistry, 2020. **63**(17): p. 9912-9927.

5. Tear, W.F., et al., *Selectivity and Physicochemical Optimization of Repurposed Pyrazolo[1,5-b]pyridazines for the Treatment of Human African Trypanosomiasis.* Journal of Medicinal Chemistry, 2020. **63**(2): p. 756-783.

6. Klug, D.M., et al., *Evaluation of a class of isatinoids identified from a high-throughput screen of human kinase inhibitors as anti-Sleeping Sickness agents.* PLOS Neglected Tropical Diseases, 2019. **13**(2): p. e0007129.

7. Klug, D.M., et al., *Lead Optimization of 3,5-Disubstituted-7-Azaindoles for the Treatment of Human African Trypanosomiasis.* Journal of Medicinal Chemistry, 2021. **64**(13): p. 9404-9430.

8. Bachovchin, K.A., et al., *Improvement of Aqueous Solubility of Lapatinib-Derived Analogues: Identification of a Quinolinimine Lead for Human African Trypanosomiasis Drug Development.* Journal of Medicinal Chemistry, 2019. **62**(2): p. 665-687.

9. Singh, B., et al., *Chemical Optimization of CBL0137 for Human African Trypanosomiasis Lead Drug Discovery* Journal of Medicinal Chemistry, 2022.
